# High-throughput 3D tracking of bacteria on a standard phase contrast microscope

**DOI:** 10.1038/ncomms9776

**Published:** 2015-11-02

**Authors:** K.M. Taute, S. Gude, S.J. Tans, T.S. Shimizu

**Affiliations:** 1 FOM Institute AMOLF, Science Park 104, 1098 XG Amsterdam, The Netherlands

## Abstract

Bacteria employ diverse motility patterns in traversing complex three-dimensional (3D) natural habitats. 2D microscopy misses crucial features of 3D behaviour, but the applicability of existing 3D tracking techniques is constrained by their performance or ease of use. Here we present a simple, broadly applicable, high-throughput 3D bacterial tracking method for use in standard phase contrast microscopy. Bacteria are localized at micron-scale resolution over a range of 350 × 300 × 200 μm by maximizing image cross-correlations between their observed diffraction patterns and a reference library. We demonstrate the applicability of our technique to a range of bacterial species and exploit its high throughput to expose hidden contributions of bacterial individuality to population-level variability in motile behaviour. The simplicity of this powerful new tool for bacterial motility research renders 3D tracking accessible to a wider community and paves the way for investigations of bacterial motility in complex 3D environments.

## Abstract (web summary)

Microscopy techniques used to study the movement of swimming microbes are limited to two dimensions or require sophisticated devices. Here, Taute *et al*. present a simple method for high-throughput 3D tracking of bacteria using standard phase contrast microscopy.

## Graphical abstract

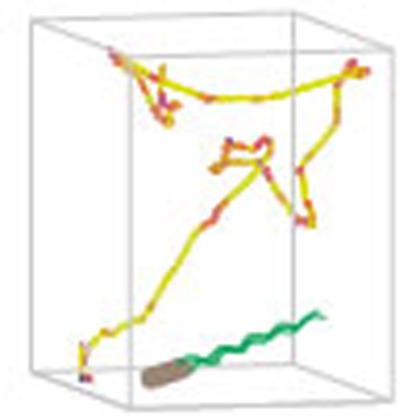

Natural habitats of swimming bacteria include complex 3D environments such as oceans, soil and tissue. They navigate these terrains by 3D motility patterns often characterized by stochastically alternating periods of linear movement (‘runs') and sudden reorientation (for example, ‘tumbles' for *Escherichia coli*, or ‘flicks' and ‘reversals' for *Vibrio alginolyticus*). These stochastic patterns are only apparent from individual trajectory data and are not accessible from population-level measurements such as bacterial number densities. Bacterial trajectories can yield rich information, for instance on the mechanism of chemotaxis^1^, the underlying signal processing^2^, interspecies differences in motility patterns^3^,^4^ and behavioural individuality^5^,^6^, which may have important ecological ramifications^7^.

Observations of bacterial motility are generally undertaken in bulk fluid to avoid artefacts arising from surface interactions^8^,^9^,^10^,^11^. As conventional light microscopy techniques only allow for localization in 2D, they capture either a projection of the 3D trajectories^12^ or the small subset that lies within the microscope's focal plane^3^,^13^. Projection implies a loss of information that can lead to ambiguities in the interpretation of trajectories (see Fig. 1). For instance, a stationary bacterium cannot be distinguished from one moving perpendicular to the plane of observation, and a right angle turn can appear as any magnitude from 0° to 180° in projection. Restricting observations to the focal plane (‘2D slicing') prevents such ambiguities, but severely limits the numbers and lengths of trajectories to a biased subset. For example, a bacterium initially swimming in plane will remain in plane if it reverses, but most likely not after a right angle turn. Similarly, longer runs have a larger chance of exiting the focal plane than short ones, leading to biases in measured run length distributions. Some of these problems are summarized in Fig. 1, and Supplementary Discussion 1 provides an in-depth analysis. In short, neither 2D projection nor slicing allow for an accurate characterization of motility patterns. Moreover, 2D observations do not fully capture motility in complex 3D environments like those encountered in nature.

Few techniques are available for tracking bacteria in 3D. Physical tracking, consisting of automatically moving the sample to keep one individual bacterium in focus^14^,^15^, has long been the gold standard of bacterial 3D tracking. This technically demanding method requires a specialized experimental set-up, and its limitation to observing only one cell at a time constrains its efficiency for characterizing the full repertoire of motile behaviours in a population. A higher throughput can be achieved by extracting 3D positions from 2D microscopy images that show multiple bacteria, but existing approaches, consisting of digital holographic microscopy and a number of defocussed imaging methods, exhibit a trade-off between performance and ease of use. Digital holographic microscopy, on the one hand, allows 3D imaging at high speed, but is not accessible to most microbiological laboratories because its application to bacteria is technically demanding and requires a customized experimental set-up^16^,^17^,^18^,^19^. Current defocussed imaging methods, on the other hand, offer technical simplicity by using the diameter of the largest observed diffraction ring as a linear measure of an object's distance 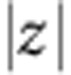 from the focal plane^20^. Implementations based on fluorescence microscopy, however, incur severe constraints in spatio-temporal resolution due to photon limitations^21^, while those using darkfield^22^ and phase contrast microscopy^23^ suffer from a significantly decreased *z* range.

Here we relieve the trade-off between performance and technical simplicity that governs existing 3D bacterial tracking methods. We increase the *z* range accessible to high-throughput tracking in defocussed phase contrast microscopy by an order of magnitude by exploiting the full complex diffraction patterns visible for objects at a given position *z* along the optical axis. Using image cross-correlations, we compare the observed patterns with a library of reference images, and assign a *z* position based on the identity of the best match. Our conceptually simple novel technique allows for the 3D tracking of any micron-sized bacteria on a standard biological phase contrast microscope at high throughput and micron-scale resolution over a *z* range of 200 μm, and at a temporal rate that is limited only by the detector readout speed.

We demonstrate the power of our approach by harnessing its throughput, range and precision to reveal substantial and hitherto unrecognized contributions of cell individuality to population-level variability in two canonical examples of bacterial motility: (i) the run-tumble behaviour of *E. coli*, and (ii) the run-reverse-flick behaviour of *V. alginolyticus*.

## Results

### 3D localization by cross-correlation to a reference library

To create a reference library showing how intensity patterns in phase contrast microscopy change with *z* position, we align and combine *z* stacks of many 1 μm silica beads (Fig. 2a). In the absence of aberrations, the intensity pattern is symmetric about the focal plane, leading to ambiguity in the sign of the *z* position^23^. We avoid such ambiguity by introducing spherical aberrations that break the symmetry and enable a unique mapping between observed pattern and *z* position.

Supplementary Movie 1 shows an example recording of swimming *E. coli* bacteria. We localize bacteria in *z* by computing the normalized image cross-correlation for the bacterial image (Fig. 2c) with images of the reference library and identifying the reference image with the highest correlation value (Fig. 2b and Supplementary Movie 2, see Methods for details). Its position in the reference library yields the corresponding *z* position. The (*x*, *y*) position is identified as the lateral shift between the two images that maximizes the cross-correlation value. Using always the last known position as a reference to constrain the search range, we repeat this procedure for each frame in a movie to assemble trajectories like the one shown in Fig. 2d, and for each of the tens of bacteria in the field-of-view.

Being based on phase contrast microscopy, our method is broadly applicable to any approximately micron-sized bacteria. We have tested the method on a range of enteric, soil and marine bacteria and recover their characteristic motility patterns (see Fig. 3a). Run-tumble motility is observed for the enteric species *E. coli* and *Salmonella typhimurium*, as well as the soil bacterium *Bacillus subtilis*, and we confirm the recently reported run-reverse-flick motility for the marine species *V. alginolyticus* and *Pseudoalteromonas haloplanktis*^3^. For the soil bacterium *Pseudomonas putida*, we observe reversals, pauses and two different run speeds, concurrent with previous reports^24^,^25^.

Using a × 40 objective, we are able to track dozens of bacteria over a range of 200 μm in *z* and at least 350 × 300 μm in *x* and *y*. As research goals differ in the minimum trajectory durations needed, we characterize the throughput as a function of this requirement using the example of *E. coli* (Fig. 3b). We observe a bimodal distribution of median trajectory speeds (see Fig. 3b inset), and attribute the lower peak to diffusing immotile bacteria. At the densities of OD_600nm_=0.003–0.006, we typically employ, we observe an average of ∼24 motile bacteria per frame whose trajectories are longer than 3 s. For comparison, Berg and Brown's^1^ benchmark study on physical 3D tracking of *E. coli* was based on 20 min of total trajectory time, which can be obtained in less than a minute using our technique.

Diffraction ring overlaps between bacteria can in principle lead to trajectory loss for one or both of the bacteria if no reference image matching the observed pattern can be identified. The typical length of trajectories is therefore constrained not only by the spatial tracking range, but also by the density of bacteria. In practice, however, our cross-correlation-based tracking technique is robust to most such encounters (see Supplementary Movie 2). For *E. coli* in the above density regime, we obtain average trajectory durations between 3 and 5 s, that is, long enough to contain several tumble events per individual, given tumble frequencies on the order of 1 s^−1^ (ref. 1).

We assess the spatial resolution of our technique by determining the root mean square (r.m.s.) localization error for tracked *z* stacks of immobilized glass beads and *E. coli* bacteria (Supplementary Figs 1 and 2, see Methods for details). For both, we obtain approximately micron-scale resolution in *z* (Fig. 3c), that is, smaller than the size of the bacteria. The lateral localization error is <0.5 μm (Fig. 3c). A frame rate of 10–30 Hz is adequate for determining key motility parameters for the species we have examined, but our technique is capable of a temporal resolution constrained only by the detector's readout speed (in our case, 100 Hz).

### Directional persistence in *E. coli* run-tumble motility

We validate our technique by applying it to the *E. coli* strain AW405, whose run-tumble motility was the first to be studied by 3D tracking in 1972 (ref. 1). Here we combine 6 data sets of 100 s each, obtained at 15 Hz, and identify runs and tumbles (Fig. 4a) in 14,188 s of total trajectory time from 2,551 motile bacteria with a minimum trajectory duration of 3 s. We employ a tumble detection algorithm based on ref. 26 but modified to master the broad range of swimming speeds (see Fig. 3b insert) found in the population (see Methods for details).

We find approximately exponentially distributed run and tumble durations (Fig. 4b) with characteristic time scales 0.64 s and 0.19 s, respectively, that are similar to those observed previously^1^. For the turning angles, we find a population mean of 57°, smaller than the 68° previously reported^1^. We also observe a significantly higher average swimming speed of 40 μm s^−1^ than the previously reported 14 μm s^−1^ (ref. 1), which we attribute to differences in growth or motility media compositions (see Supplementary Discussion 3). Furthermore, we likely sample the population more broadly with respect to swimming speeds as physical tracking was only applied to a manually selected set of swimming bacteria and was incapable of following the fastest individuals in the population^26^. To determine whether the speed range of the bacterial population affects the observed turning angles, we exploit the high throughput of our method and split up our data set of 8,058 turning angles to create separate turning angle distributions for 5 different run speed ranges. Surprisingly, we find that the turning angle distribution varies with run speed (Fig. 4c). Both theoretical considerations^27^,^28^,^29^ and experimental evidence^12^,^13^ indicate that the average cosine of the turning angle, called directional persistence, is an important determinant of chemotactic performance. We show that the directional persistence increases approximately linearly by a factor of 3 over the speed range of 20–60 μm s^−1^ (Fig. 4d). As the tumble frequency we obtain is independent of run speed (Supplementary Fig. 3), we exclude detection bias as a source of this correlation. The increased persistence of faster swimming bacteria is also evident upon visual inspection of trajectories (Supplementary Fig. 4).

Directional persistence is expected to have opposite effects on the conflicting chemotactic requirements of drift velocity and accumulation at attractant concentration peaks^27^, hence its dependence on run speed could have non-trivial consequences for chemotactic behaviour. Elucidating how these opposing constraints are balanced in run-tumble motility could yield clues to the selection criteria relevant in the evolution of *E. coli* chemotaxis.

In addition, our observation may help shed light on the mechanisms underlying reorientation during a tumble. *E. coli* swimming is driven by the rotation of helical appendages called flagella. While during runs, all flagella rotate counterclockwise and form a bundle that pushes the cell forward, a tumble occurs when one or more flagella reverse direction and leave the bundle^30^. The resulting reorientation has been modelled as rotational diffusion, which implies a persistence that decreases exponentially with tumble duration^31^. In contrast, the modulation in turning angle we observe does not result from differences in tumble duration (Supplementary Fig. 3). Propulsion and reorientation with multiple flagella are challenging to model, and even the mechanical determinants of swimming speed remain unknown^32^. Further imaging studies of swimming cells with labelled flagella^30^ may be able to clarify this point. Our findings then provide strong constraints for possible hydrodynamic models of flagellar bundle mechanics.

### Behavioural individuality in run-reverse-flick motility

**Figure 1:**
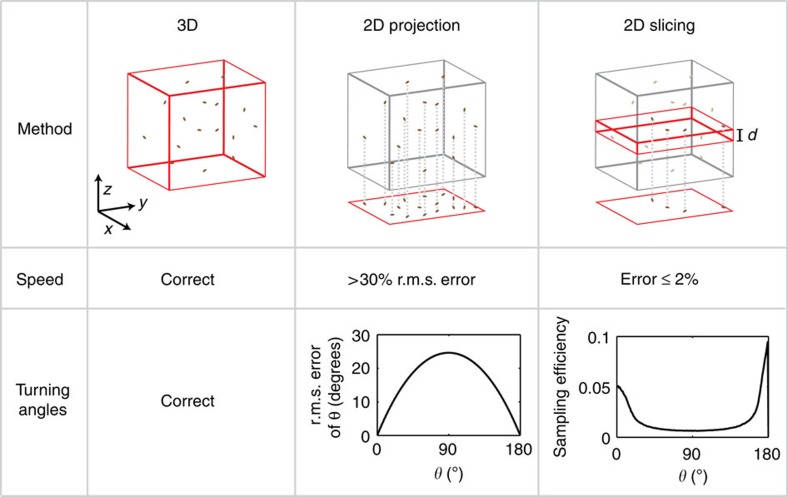
Comparison of 3D and 2D tracking methods. In 3D tracking (left column), full position information is obtained, and speed and angle measurements are correct aside from the effects of localization errors and acquisition frequency which we neglect here. 2D projection (centre column) of the same volume introduces systematic errors in speed and angle measurements. In 2D slicing (right column), observations are constrained to a thin focal plane of thickness *d*. If we assume that runs have to lie fully within the slice and are five times longer than *d*, corresponding to typical parameters in the observation of *E. coli* and many other bacteria, measurement errors are minimized. However, the vast majority of turning events within the focal plane are rejected, with a bias against angles near 90°. Supplementary Discussion 1 provides derivations for the data presented here.

Aside from the advantages shown in Fig. 1, 3D tracking is superior to 2D slicing with respect to the trajectory durations that can be measured before an individual is lost. Here we show how this feature can be harnessed to reveal phenotypic heterogeneity in motile behaviour using the example of the marine bacterium *V. alginolyticus*.

Our 3D trajectories confirm the run-reverse-flick behaviour recently reported by Xie *et al*.^3^ as composed of runs interrupted by turning events that alternate between reversals (180°) and ‘flicks' of a smaller magnitude (Fig. 5a,b). Flicks were recently shown to result from buckling of the flagellar hook when bacteria resume forward motion after a reversal^4^ (see Fig. 5c). The broadness of the flick angle distribution obtained by Xie *et al*.^3^ in 2D at the population level leaves open the question whether the flick process is inherently random or whether individuals differ in their flick behaviour.

Our large *z* range enables us to obtain many trajectories that contain a sufficient number of flicks to test the hypothesis of inter-individual variability. While the broad population distribution of flick angles we extract (Fig. 5d) is similar to that observed previously in 2D slicing^3^, we show that individuals actually show very narrow flick angle distributions with different means (Fig. 5e). Moreover, we find that the flick angle, which is defined as the angle between the reverse and forward runs flanking the flick event (Fig. 5c), shows a pronounced dependence on body length (Fig. 5e). The observed dependence is consistent with longer bacteria experiencing an increased viscous drag that counteracts the angular reorientation during a flick^4^, leading to a flick angle closer to 180°. Comparing the flick angle s.d. of 32° on the population level and ∼15° for individuals, we conclude that differences between individuals account for ∼80% of the observed population variance.

Based on the previously measured population mean flick angle of ∼90 degrees (ref. 3), models of run-reverse-flick motility have thus far assumed a complete randomization of the swimming direction during flicks^33^,^29^. In contrast, our data reveal a significant level of determinism in the flicking behaviour of individuals that may have implications for the performance of run-reverse-flick motility in chemotaxis. In addition, the observed behavioural individuality implies a spread in motility patterns even within isogenic populations which may have important ecological consequences. Furthermore, our result suggests that individuals might undergo a systematic shift in motility patterns during their life cycle as they elongate between cell divisions.

## Discussion

We report a simple yet powerful method for high-throughput tracking of bacteria in 3D, which stands out among existing techniques in its ease of use and broad applicability. No components beyond a standard phase contrast microscope and a camera are required for the data acquisition, and any micron-sized bacteria can be tracked. In contrast to physical tracking methods that follow single cells^14^,^15^, dozens of trajectories can be acquired simultaneously. Relative to previous defocused imaging methods^21^,^22^,^23^, our enhanced tracking range in *z* enables acquisition of significantly longer 3D trajectories for unconfined cells. The micron-scale resolution we present in the current implementation is adequate for most bacterial tracking applications, but even higher resolution can be achieved by trading off tracking range in favour of resolution. For instance, over a range of 40 μm in *z*, a *z* error <250 nm is achievable by removing the deliberately introduced spherical aberrations (Supplementary Discussion 2 and Supplementary Fig. 5).

The advantages of 3D tracking extend beyond precise measurements of turning angles. Accurate determination of run length statistics requires both unambiguous detection of turning events (which is challenging in 2D projection) and the acquisition of trajectories significantly longer than typical run lengths (which rarely occur in 2D slicing) to avoid sampling biases. For example, assuming an underlying exponential run length distribution with parameters typical for *E. coli*, we estimate that 2D slicing incurs an error by a factor of at least 2 in the measurement of the average run length, whereas our 3D tracking underestimates the value by only 10–20% (Supplementary Discussion 1 and Supplementary Fig. 6). 3D tracking combines the ability to acquire longer trajectories with a high precision of turning angle determination, and both of these were essential for our discovery of individuality in *V. alginolyticus* flicking behaviour (Fig. 5). In 2D, long trajectories can only be achieved by either confining the bacteria to quasi-2D chambers^34^, where surface interactions perturb their behaviour^8^,^10^,^11^, or by observing unconfined bacteria in 2D projection, where the ability to detect individuality in turning angles is severely hampered by the increased measurement errors incurred in projection. We estimate that, to distinguish *V. alginolyticus* individuals with the same certainty in 2D projection as in 3D tracking, at least three times as many flick events per individual need to be observed, which, given the low frequency of long trajectories, would require orders of magnitude more data to be acquired (Supplementary Discussion 1). Thus the precise angle measurements obtained by 3D tracking reduce the number of events required to detect differences between individuals to routinely achievable values.

The high throughput of our method enables rapid and quantitative characterization of single-cell motility patterns across large populations. The applications demonstrated here highlight how the resulting large data sets can be used to analyse the contributions of stochasticity and individuality in bacterial behaviour, as well as gain insight into the underlying mechanisms. Such phenotypic heterogeneity in motile behaviour may represent a bet-hedging strategy with significant ecological implications^5^,^7^. To predict the consequences of single-cell behaviours over time and their combined effect at the population level, it is crucial to differentiate between systematic cell-to-cell variation and temporal randomness. In practice, it can be challenging to distinguish stochastic processes from deterministic ones governed by hidden variables, such as cell attributes that are not directly measured in the experiment^35^. With sufficiently large data sets, however, correlations between measured parameters can reveal the presence of such hidden variables. Our discovery of a correlation between run speed and directional persistence in *E. coli* motility exemplifies how our method's high throughput can be exploited to reveal hidden deterministic factors and place limits on the magnitude of stochastic contributions to motile behaviour. More generally, our new method enables a data-driven approach towards a mechanistic understanding of randomness and diversity in bacterial motility behaviour.

Bacteria make up a significant fraction of the biomass on our planet^36^, and the impact of their motility on human health^37^,^38^, the environment^39^,^40^, agriculture^41^ and material processing^42^ is increasingly recognized in medicine, ecology and industry. Our method is ideally suited to service the growing interest in the study of bacterial motility across fields, as it enables accurate and efficient characterization of bacterial motility without specialized equipment. Because it does not require any labelling of bacteria, it is applicable to laboratory strains and natural/clinical isolates alike. Since the technique is compatible with any assay amenable to phase contrast microscopy, it opens the doors to fully capturing bacterial motility in a broad range of contexts from microfluidic platforms to complex 3D environments like those encountered in nature.

## Methods

### Bacterial culturing

Overnight cultures of bacteria were inoculated from glycerol stocks stored at −80 °C. Day cultures were inoculated with 40 μl overnight culture in 10 ml growth medium and grown in 100 ml culture flasks being shaken at 200 r.p.m. Table 1 lists the bacterial strains, culture media and temperatures, and Table 2 the media compositions. Bacteria were harvested from the day cultures in mid-exponential phase. For dissimilar growth and motility medium, bacteria were decanted into 50 ml plastic tubes and washed by three rounds of centrifugation (5 min at 5 k r.p.m. at 21° C in an Eppendorf 5804 R centrifuge), each followed by gentle resuspension in 10 ml motility medium. Bacteria were used for imaging within 2 h after washing.

### Bacterial sample preparation

Sample chambers with a height of ∼300 μm were created by using three layers of parafilm as a spacer between a microscopy slide and an 18 × 18 mm #1 coverslip (Menzel Gläser, thickness 150±3 μm). The chamber was heated on a hotplate and pressed to seal. Bacteria were diluted to a target optical density of 0.003–0.006 at 600 nm in the appropriate motility medium and flowed into the chamber via capillary forces. The ends of the chamber were then sealed with hot valap (a mixture of vaseline, lanolin and paraffin).

### Microscopy and data acquisition

The sample chamber was placed on a Nikon TE2000-U inverted optical microscope equipped with an air condenser (Nikon LWD, numerical aperture (NA) 0.52) and a × 40 phase contrast lens (Nikon, S Plan Fluor ELWD, × 40, Ph2, NA 0.6). We set the objective lens's correction collar to 1.2 mm to introduce spherical aberrations, which introduce asymmetry about the focal plane in the point spread function and avoid localization problems near the focus. As an alternative to using the correction collar, spherical aberrations can also be introduced by imaging through a thicker substrate than the objective lens is optimized for, for example, a slide instead of a coverslip. We verified that the point spread function is robust to day-to-day changes in the optical set-up as may arise from temporary removal of components such as camera and objective lens, readjustments of movable parts such as field stops, small errors in the correction collar setting and the variation of coverslip thickness within one package, (± 3μm). For recordings, we moved the sample 100 μm towards the objective lens relative to the chamber's inner bottom surface, corresponding to a focal position 133 μm inside the sample, given the refractive index of *n*_MotM_=1.334 of our motility medium. The illumination was adjusted to produce ∼20,000 counts per pixel. For each recording, 1,500 frames were acquired at a frame rate of 15 Hz and an exposure time of 5 ms using a PCO.edge sCMOS camera (pixel size 6.5 μm, 2,560 × 2,160 pixels). The data were saved as a series of 16-bit tiff files of 193 images each, corresponding to a maximum file size of 2 GB.

### Background correction

To remove unwanted background features, we adopt a median-based correction technique. For each movie file, we compute the pixel-wise median of all contained images. We then divide each image by this median and multiply by 20,000 to approximately restore the original mean intensity. This procedure removes all stationary objects and creates a flat background.

### Image cross-correlation

The normalized cross-correlation of two image matrices *I*_1_ and *I*_2_ is given by


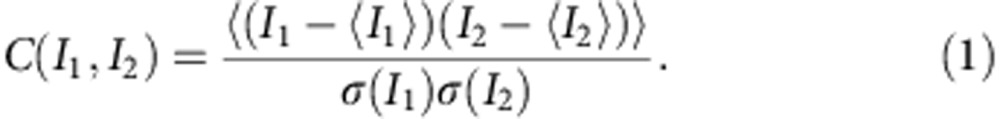


Here 
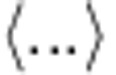
 and *σ*(...) denote the mean and s.d. across all pixels. In practice, images are subject to noise:


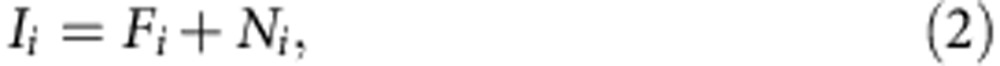


where *N*_*i*_ refers to the noise and *F*_*i*_ to the feature of interest. What is generally of interest is a measure of the similarity of the noisefree features of two images, *C*(*F*_1_, *F*_2_). Inserting equation (2) into equation (1), and assuming that the noise has zero mean and is uncorrelated with features or between different images, we find


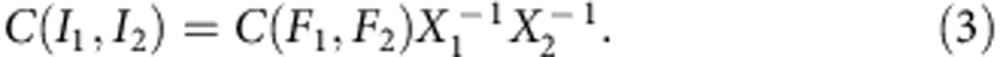


which allows a conversion from the readily computed *C*(*I*_1_, *I*_2_) to the desired *C*(*F*_1_, *F*_2_) via the correction factors


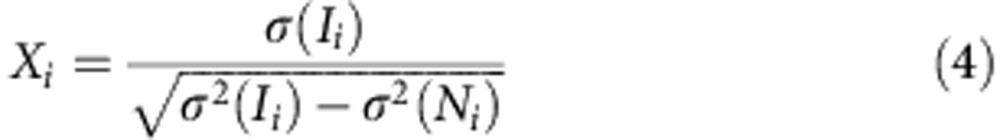


which can be computed if the variance of the noise, *σ*^2^(*N*_*i*_), is known. For motility movies, we estimate a fixed value for the noise variance based on the variance of the background by measuring the inter-pixel intensity variance in areas of the sample that show no features, but only background. For the reference library, the noise varies between images as they differ in the amount of data contributing to them. We therefore estimate the noise separately for each image of the reference library.

Image cross-correlations are computed via fast Fourier transforms on regions of interest of 256 × 256 pixels. The output is a matrix whose indices give the cross-correlation values for each possible lateral shift of the images against each other.

### Reference stack generation

Reference stacks were created from images of spherical beads rather than bacteria, which are generally elongated and oriented in random directions. The symmetry of the beads eases alignment of different stacks for averaging, and prevents a bacterium's orientation in the *x*–*y* plane from affecting its cross-correlation to the reference image. Glass beads with a nominal diameter of 1 μm (PSi-1.0, Kisker Biotech, Germany) were empirically determined to produce contrast similar to bacteria in phase contrast microscopy. To facilitate image acquisition at defined positions relative to the focus with a surrounding medium of refractive index similar to media used in motility assays, the beads were immobilized in a 3% polyacrylamide (PAC) gel. The refractive index of the gel at this concentration is *n*_PAC3_=1.338, comparable to *n*_MotM_=1.334 measured for motility medium. Refractive index measurements were performed with a hand-held refractometer calibrated against sucrose solutions of known concentrations. Lower concentrations of acrylamide were found not to solidify. From a 50 μg ml^−1^ stock solution, the beads were diluted 1:100 into an acrylamide solution (375 μl 40% acrylamide, 4.57 ml H_2_O, 50 μl 10% ammonium persulfate in H_2_O, 5 μl N,N,N′,N′-tetramethylethane-1,2-diamine) which was then flowed into a sample chamber as described above and sealed with nail polish. Once the PAC solution had solidified, image stacks were acquired of the now immobilized glass beads. Focal plane and imaging conditions were as for live bacteria. Using a piezo stage with a range of 200 μm, the sample was moved in 1,000 steps of Δ*x*=100 nm, centred about the focal plane, and one image acquired at each position. These steps correspond to steps in focal plane by *n*Δ*x*=134 nm inside the sample.

For each bead localized inside the scanned volume, a substack of 512 × 512 pixels in *x* and *y* approximately centred laterally about the bead was extracted. The substacks were background-corrected based on the median across the entire substack.

As beads were located at unknown random positions in *x*, *y* and *z*, their image stacks need to be aligned relative to each other before being combined. 3D cross-correlations can be employed to determine their 3D positions relative to each other. For each bead *k*, a 3D array *B*^*k*^ consisting of the inner 256 × 256 pixel region of its image stack is created. For one arbitrary, but fixed bead *i* and each other bead *j*, the normalized 3D cross-correlation, *C*^*ij*^, of *B*^*i*^ and *B*^*j*^ is computed. *C*^*ij*^ is another 3D array, with each entry representing the cross-correlation value of the arrays *B*^*i*^ and *B*^*j*^ at one particular 3D shift relative to each other. The position of the entry with the maximum value in *C*^*ij*^ then reflects the 3D relative shift of the beads *i* and *j* to each other.

With known relative 3D alignment, the full 512 × 512 stacks could be combined into one reference library. Because the initial placement of the beads was random across *z*, the substacks cover different ranges relative to the bead's focus. Conversely, not every bead stack covers every possible *z* position relative to the bead's focus. For each *z* position relative to the focus, an image was obtained as the pixel-wise median across the respective images from those substacks covering this *z* position. Only those positions with at least 15 contributing substacks were retained. The resulting image stack covered 220 μm and was constructed from image stacks of 73 different beads.

### Tracking algorithm

Tracking was implemented using a custom-written Matlab (The MathWorks) programme. Briefly, once a bacterium is identified, it is tracked both forwards and backwards in time, always using the last known position to constrain the search radius in all three spatial dimensions.

Positions are determined in a multistep procedure. A 256 × 256 pixel region-of-interest around the last known position is cross-correlated against a substack of the reference library centred about the last known *z* position. For each reference image in the substack, the maximum cross-correlation value across a range of lateral shifts is determined. The search range is set to cover the maximal displacement possible at the maximum plausible bacterial swimming speed, which we estimate as 150 μm s^−1^ for the marine bacteria and 100 μm s^−1^ for the other species.

A 13-point neighbourhood around the maximum cross-correlation value is fitted with a second order polynomial to extract the *z* position of the maximum. The position of the parabola's maximum is rounded to the *z* position of the nearest reference image, which is taken as the object's *z* position. The new lateral position is determined from the lateral shift at which the cross-correlation with the chosen reference image is maximal. If the cross-correlation value at the determined position is lower than a retention threshold (typically set to 0.35), the trajectory ends. This can occur when the diffraction pattern of the tracked bacterium overlaps with another one with higher contrast. Positions are also rejected if they lie at the edge of the search range. This typically occurs when bacteria leave the tracking range.

For each frame, putative new bacteria are identified using a thresholding procedure. Briefly, the absolute difference to the image median is computed, and a threshold applied to create a binary image. Typical threshold values used are 0.03–0.1 times the image median. The binary image is eroded to avoid spurious noise, followed by dilation to fill in gaps between diffraction rings. Disks with radii of 3 pixels and 30 pixels are used as structuring elements for erosion and dilation, respectively. The resulting image is segmented, and the centroids of segments are taken as putative lateral bacterial locations. Locations within a threshold lateral distance of 60 pixels to known bacteria or 130 pixels to the image border are rejected. These distances are set deliberately large so as to accommodate the uncertainty of the putative locations.

For each remaining putative location, the reference library is searched for a matching image. The detection procedure is not sensitive enough to identify bacteria throughout the entire tracking range, therefore we constrain the search to approximately the inner 60% of the reference library and thus miss trajectories that never enter this range. If a correlation with a reference image above an acceptance threshold (typically set at 0.6) is found, a new trajectory is created if the position does not lie within a joining distance to a known bacterium. We typically use a joining distance of 10 pixels in *x* and *y* and 30 reference library slices with a spacing of 134 nm in *z*. Upon creation, the bacterium is tracked backwards in time. If its position at a frame in the past lies within the joining distance of the position at which another trajectory ended at the same frame, the trajectories are joined.

Our custom Matlab-based implementation of the tracking algorithm is available on request. A user-friendly version is under development for release at a later date.

### Determination of localization precision

To determine the localization precision of our technique for bacteria, we prepared stacks for immobilized *E. coli* as for glass beads above, but with either 100 or 200 nm steps between frames. The tracking procedure was then applied to stacks of glass beads and bacteria. For glass beads, a rotated version of the reference library (180° about the *z* axis) is used to avoid artefacts from spurious correlations due to the fact that the reference library was created from these same stacks of 73 glass beads. For *x* and *y*, localization errors were determined as s.d. of the lateral position. For *z*, localization errors are determined as the residuals of a linear fit to the extracted *z* position against the known *z* position. As the magnitude of these errors depends on the *z* position, we compute the r.m.s. error across 48 different bacteria for bins with a width of 2 μm in *z* (Fig. 3c).

### *E. coli* run-tumble analysis

From the trajectories obtained from our tracking procedure, velocities are computed as the fourth order central difference^26^,


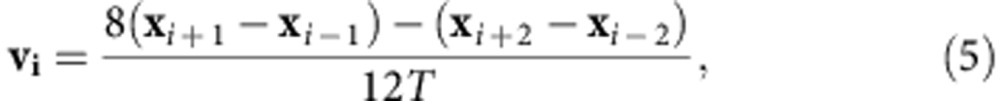


where *T*=1/15 s is the time between frames, and direction changes 
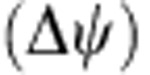
 as the angles between subsequent velocity vectors.

We only consider trajectories that span at least 3 s and show a median speed larger than 17 μm s^−1^. Our run-tumble calling algorithm follows the procedure laid out by ref. 26 in that a run ends at time point *t*_*i*_ if either 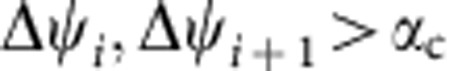 or if 
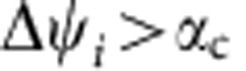
 and the angle between the sum of the velocities from *t*_*i*−2_−*t*_*i*_ and those from *t*_*i* +1_−*t*_*i*+3_ also exceed *α*_c_. A new run begins at *t*_*i*_ if 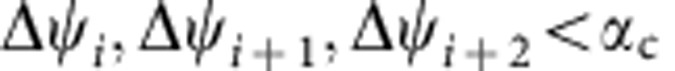. A run hence has a minimal duration of four time points, while a tumble can have zero length.

To cope with the large range of swimming speeds present in the bacterial population, the threshold *α*_c_ is determined relative to the median direction change, 
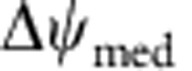
, each individual displays during runs: 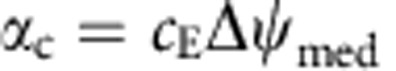, as opposed to using a fixed absolute value as previously^26^. To arrive at a self-consistent definition of runs, we iterate this procedure 20 times. The number of tumbles found typically stabilizes by about 10 iterations. The factor *c*_E_ was chosen so as to balance the rate of missed tumbles and erroneously called tumbles based on visual inspection of trajectories. At the chosen value of *c*_E_=3, we observe ∼5% false negatives and 5% false positives.

Turning angles are measured as the angle between the sum of the last three velocity vectors of the run preceding the tumble and the first three of the subsequent run.

### *V. alginolyticus* run-reverse-flick analysis

For *V. alginolyticus*, we only consider trajectories that span at least 3 s and show a median speed larger than 40 μm s^−1^ as flicks are rare at low speeds^4^. The average speed of motile bacteria is 48 μm s^−1^. To detect turning events, we employ a similar strategy as for the tumble detection for *E. coli*, with several modifications. First, rather than the iterative method used for *E. coli*, we fix the detection threshold at 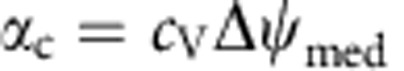 with *c*_V_=4. Second, a new run begins at *t*_*i*_ if 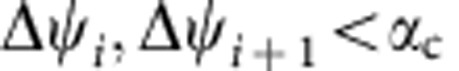, hence runs can be as short as three time points. And third, we measure turning angles between sums of two rather than three velocity vectors.

Among these, we then identify flicks and reversals in a two-step procedure. First, if all even turning events within a trajectory with at least six events are at least 2 s.d away from the mean of the odd turns, and *vice versa*, then the set with the smaller mean is considered flicks and the other one reversals. If the odd and even turns are not well-separated, then if a turn has a magnitude below a threshold and both adjacent turns are above it, the middle turn is considered a flick and the adjacent ones reversals. Using a threshold of 155°, we identify 581 flicks and 771 reversals out of 2,180 turns detected in more than 30 min of total trajectory time.

For a set of 20 trajectories that contained at least 6 flicks, body length was measured from frames where the bacterium was swimming approximately in the *x*–*y* plane. Due to the spherical aberrations present, the bacterium's body is visible for a range of *z* positions. We estimate an error of 2 pixels for the length measurement.

## Additional information

**How to cite this article:** Taute, K. M. *et al*. High-throughput 3D tracking of bacteria on a standard phase contrast microscope. *Nat. Commun.* 6:8776 doi: 10.1038/ncomms9776 (2015).

## Supplementary Material

Supplementary Information Supplementary Figures 1-6, Supplementary Discussion 1-3 and Supplementary References

Supplementary Movie 1 A 21.5 s long segment of a recording of swimming *E. coli* bacteria, showing 37.5% of the full field of view. Playback is real time. The video has been binned 5x5 times spatially. Scale bar: 50 μm.

**Figure 2:**
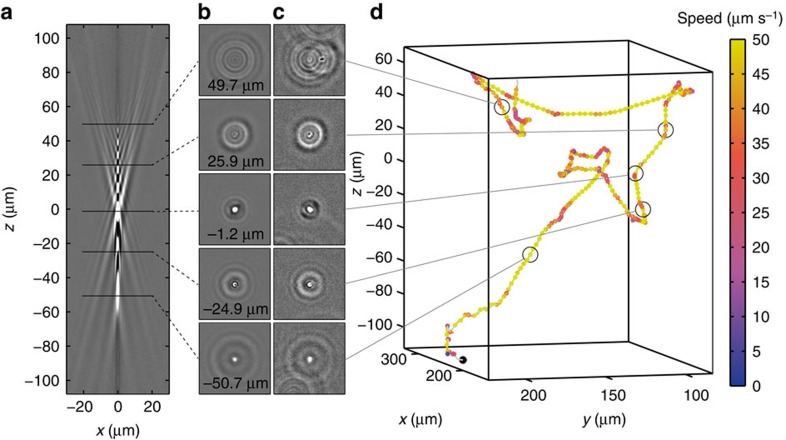
Tracking bacteria in 3D by comparing their out-of-focus diffraction patterns to a reference library. (**a**) A vertical slice through a reference library created by combining 73 aligned image stacks obtained for 1 μm silica beads. (**b**) Horizontal slices from the reference library at positions marked in **a**. (**c**) Images of a swimming *E. coli* bacterium at the corresponding positions. (**d**) Reconstructed 3D trajectory for the bacterium in **c** (see Supplementary Movie 3 for a rotating view). The trajectory starting point is marked by a black dot.

**Figure 3:**
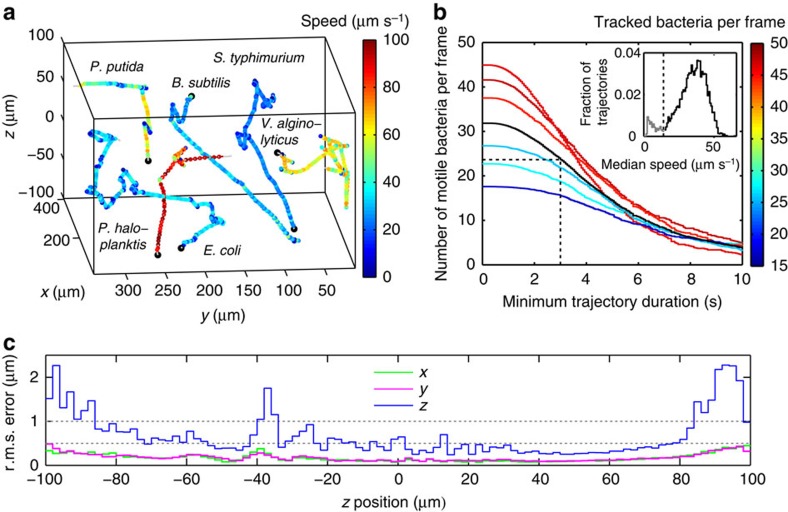
Performance of 3D tracking. (**a**) The spatial range and broadness of applicability are illustrated by a compilation of example trajectories obtained by 3D tracking of a selection of bacterial species, recorded at 15 Hz. Trajectory starting points are marked by black dots. The shown volume corresponds approximately to the tracking range. (**b**) Throughput analysis for *E. coli* tracking, showing the average number of motile bacteria (defined by a median speed above 17 μm s^−1^, see inset) per frame with a given minimum trajectory duration. Lines of different colour correspond to 100 s long recordings at 15 Hz on 6 samples with different densities (see colour map), black denotes their average. A total of 5,890 trajectories were recorded. For the run-tumble analysis in Fig. 4, we combine these data and consider only trajectories with a minimum duration of 3 s. (**c**) r.m.s. localization error in *x*, *y* and *z* across the tracking *z* range. Errors are determined by tracking *z* stacks of 48 bacteria immobilized at random positions in the sample. The average r.m.s. error is <0.7 μm in *z* and <0.2 μm in *x*, *y*.

**Figure 4:**
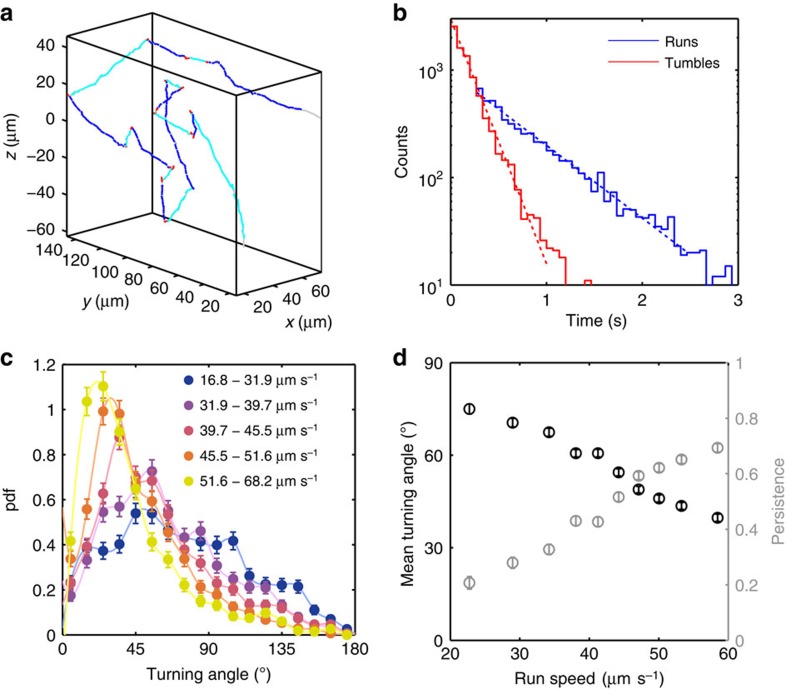
Analysis of *E. coli* run-tumble motility. The analysis is based on 6 recordings of 100 s each from 4 different days, in which 14,188 s of total trajectory time from 2,551 motile bacteria with a minimum trajectory duration of 3 s were considered. (**a**) Example trajectory with marked tumbles (red) and runs (alternating between dark and light blue). (**b**) Histogram of run (blue) and tumble (red) durations from 6,015 and 8,350 events, respectively, with exponential fit. (**c**) Turning angle distribution plotted for different ranges of median run speeds. Speed ranges are chosen to contain approximately equal total trajectory time. The total number of analysed tumbles is 8,058. (**d**) Mean turning angle (black, left axis) and directional persistence (mean cosine of the turning angle, grey, right axis) plotted against average run speed. All error bars denote the s.e.m.

**Figure 5:**
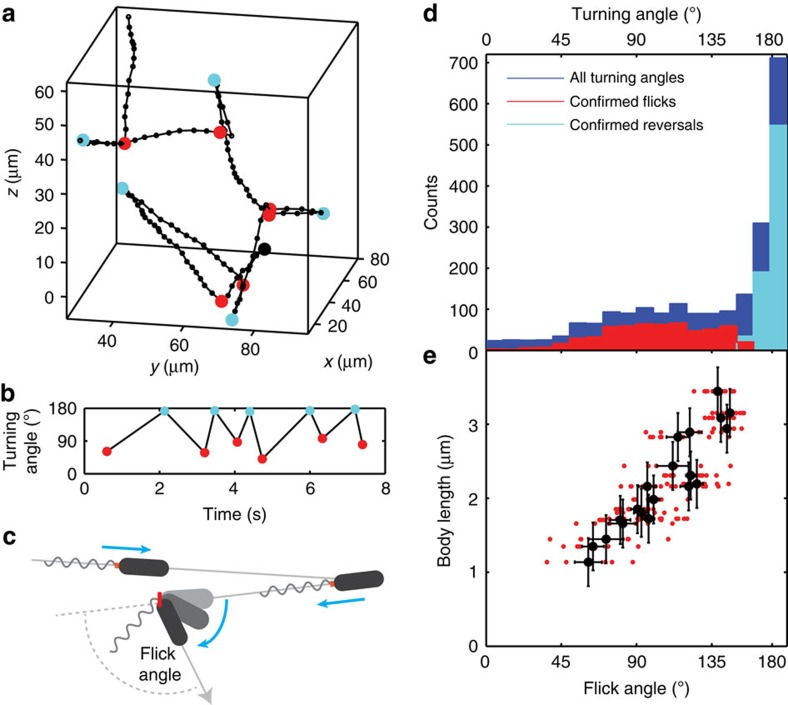
Individuality in run-reverse-flick motility. (**a**) An 8.3-s-long 3D trajectory of a *V. alginolyticus* bacterium displaying run-reverse-flick motility. Flicks (red) and reversals (cyan) are marked. (**b**) Turning angles alternate between reversals (∼180°) and smaller flicks. (**c**) The direction of rotation of the polar flagellum determines whether the bacterium is pushed or pulled by the flagellum. Reversals result from switching from pushing to pulling. Flicks result from buckling of the flagellar hook (red) on resuming forward movement. A smaller deflection corresponds to a larger measured flick angle. (**d**) Histograms showing the population distribution of all turning angles (2,180 events, dark blue), confirmed flicks (581 events, red) and confirmed reversals (771 events, cyan). (**e**) Body length and flick angles for 20 individuals that displayed at least 6 flicks in their trajectory. Black: mean±s.e.m., red: scatter plot of underlying data.

Supplementary Movie 2 Tracking one bacterium from Supplementary Movie 1, corresponding to the individual depicted in Fig. 2c,d in the main text. The left panel is centred on the last known x-y position of the bacterium. The right panel shows the best-matching reference image. The black cross in the left panel marks the current x-y position of the bacterium as determined by the 3D tracking algorithm. Scale bar: 10 μm.

Supplementary Movie 3 3D trajectory assembled for the bacterium tracked in Supplementary Movie 2, corresponding to a rotating view of Fig. 2d in the main text.

## Figures and Tables

**Table 1:** Strains and culturing conditions.

**Species**	**Strain**	***T***_**ON**_	***T***_**DC**_	**Growth medium**	**Motility medium**
*Escherichia coli*	AW405*	30 °C	33.5 °C	TB	0.18% MC/MotM
*Salmonella typhimurium*	LT2†	30 °C	33.5 °C	TB	0.18% MC/MotM
*Bacillus subtilis*	OI1085‡	30 °C	33.5 °C	BsMM	BsMM
*Pseudomonas putida*	KT2440§	30 °C	33.5 °C	TB	0.18% MC/MotM
*Vibrio alginolyticus*	YM4‡	RT	RT	MBc	TMN||
*Pseudoalteromonas haloplanktis*	ATCC 700530‡	RT	RT	MBc	MBc

BsMM, *B. subtilis* minimal medium; MBc, Marine Broth 2216 (Difco), clarified by centrifugation at 4,500*g* for 10 min; MC, (hydroxypropyl)methyl cellulose (H7509, Sigma Aldrich); MotM, *E. coli* motility medium; RT, room temperature; TB, tryptone broth; TMN, TMN motility medium; *T*_ON_/*T*_DC_, temperature of overnight/day culture, respectively.

^*^Strain provided by Howard Berg (Harvard University).

^†^Strain provided by Kelly Hughes (The University of Utah).

^‡^Strain provided by Roman Stocker (MIT).

^§^Strain provided by Carsten Beta (The University of Potsdam).

^||^Some samples contained 0.002% Tween 20 to limit adhesion of bacteria to the sample chamber surfaces.

Media compositions are provided in Table 2.

**Table 2:** Media used for growth and motility experiments.

**Medium**	**Composition**
TB	1% bacto tryptone
	0.5% NaCl
	pH 7.0
	
MBc	Difco Marine Broth 2216
	Centrifuged to remove precipitate
	
MotM	10 mM KPO_4_
	0.1 mM EDTA
	1 μM L-methionine
	10 mM lactic acid
	0.067 mM NaCl
	pH 7.0
	
BsMM	50 mM KPO_4_
	1.2 mM MgCl_2_
	0.14 mM CaCl_2_
	1 mM (NH_4_)_2_SO_4_
	0.01 mM MnCl_2_
	20 mM sorbitol
	0.02% Bacto tryptone
	50 μg ml^−1^ L-histidine
	50 μg ml^−1^ L-methionine
	50 μg ml^−1^ L-tryptophan
	pH 7.0
	
TMN	50 mM Tris-HCl
	5 mM MgCl_2_
	5 mM glucose
	300 mM NaCl
	pH 7.5

BsMM, *B. subtilis* minimal medium; MBc, Marine Broth 2216 (Difco), clarified by centrifugation at 4,500*g* for 10 min; MotM, *E. coli* motility medium; TB, tryptone broth; TMN, TMN motility medium.

## References

[b1] Berg H. & Brown D. Chemotaxis in *Escherichia coli* analysed by three-dimensional tracking. Nature 239, 500–504 (1972). 4563019 10.1038/239500a0

[b2] Ahmed T. & Stocker R. Experimental verification of the behavioral foundation of bacterial transport parameters using microfluidics. Biophys. J. 95, 4481–4493 (2008). 18658218 10.1529/biophysj.108.134510 PMC2567943

[b3] Xie L., Altindal T., Chattopadhyay S. & Wu X.-L. Bacterial flagellum as a propeller and as a rudder for efficient chemotaxis. Proc. Natl Acad. Sci. USA 108, 2246–2251 (2011). 21205908 10.1073/pnas.1011953108 PMC3038696

[b4] Son K., Guasto J. S. & Stocker R. Bacteria can exploit a flagellar buckling instability to change direction. Nat. Phys. 9, 494–498 (2013).

[b5] Spudich J. & Koshland D. Non-genetic individuality: chance in the single cell. Nature 262, 467–471 (1976). 958399 10.1038/262467a0

[b6] Masson J.-B., Voisinne G., Wong-Ng J., Celani A. & Vergassola M. Noninvasive inference of the molecular chemotactic response using bacterial trajectories. Proc. Natl Acad. Sci. USA 109, 1802–1807 (2012). 22307649 10.1073/pnas.1116772109 PMC3277171

[b7] Frankel N. W. . Adaptability of non-genetic diversity in bacterial chemotaxis. eLife 3, e03526 (2014). 10.7554/eLife.03526 PMC4210811 25279698

[b8] Frymier P. & Ford R. Three-dimensional tracking of motile bacteria near a solid planar surface. Proc. Natl Acad. Sci. USA 92, 6195–6199 (1995). 7597100 10.1073/pnas.92.13.6195 PMC41669

[b9] Berke A., Turner L., Berg H. & Lauga E. Hydrodynamic attraction of swimming microorganisms by surfaces. Phys. Rev. Lett. 101, 038102 (2008). 18764299 10.1103/PhysRevLett.101.038102

[b10] Li G., Tam L.-K. & Tang J. X. Amplified effect of Brownian motion in bacterial near-surface swimming. Proc. Natl Acad. Sci. USA 105, 18355–18359 (2008). 19015518 10.1073/pnas.0807305105 PMC2587629

[b11] Molaei M., Barry M., Stocker R. & Sheng J. Failed escape: solid surfaces prevent tumbling of *Escherichia coli*. Phys. Rev. Lett. 113, 068103 (2014). 25148353 10.1103/PhysRevLett.113.068103

[b12] Saragosti J. . Directional persistence of chemotactic bacteria in a traveling concentration wave. Proc. Natl Acad. Sci. USA 108, 16235–16240 (2011). 21918111 10.1073/pnas.1101996108 PMC3182703

[b13] Bubendorfer S., Koltai M., Rossmann F., Sourjik V. & Thormann K. M. Secondary bacterial flagellar system improves bacterial spreading by increasing the directional persistence of swimming. Proc. Natl Acad. Sci. USA 111, 11485–11490 (2014). 25049414 10.1073/pnas.1405820111 PMC4128160

[b14] Berg H. How to track bacteria. Rev. Sci. Instrum. 42, 868–871 (1971). 4940742 10.1063/1.1685246

[b15] Liu B. . Helical motion of the cell body enhances Caulobacter crescentus motility. Proc. Natl Acad. Sci. USA 111, 1–5 (2014). 10.1073/pnas.1407636111 PMC4128131 25053810

[b16] Garcia-Sucerquia J. . Digital in-line holographic microscopy. Appl. Opt. 45, 836–850 (2006). 16512525 10.1364/ao.45.000836

[b17] Vater S. M. . Swimming behavior of Pseudomonas aeruginosa studied by holographic 3D tracking. PLoS ONE 9, e87765 (2014). 24498187 10.1371/journal.pone.0087765 PMC3909247

[b18] Molaei M. & Sheng J. Imaging bacterial 3D motion using digital in-line holographic microscopy and correlation-based de-noising algorithm. Opt. Express 22, 3232–3238 (2014). 10.1364/OE.22.032119 PMC4317141 25607177

[b19] Cheong F. . Rapid, high-throughput tracking of bacterial motility in 3D via phase-contrast holographic video microscopy. Biophys. J. 108, 1248–1256 (2015). 25762336 10.1016/j.bpj.2015.01.018 PMC4375448

[b20] Speidel M., Jonás A. & Florin E.-L. Three-dimensional tracking of fluorescent nanoparticles with subnanometer precision by use of off-focus imaging. Opt. Lett. 28, 69–71 (2003). 12656488 10.1364/ol.28.000069

[b21] Wu M., Roberts J. W., Kim S., Koch D. L. & DeLisa M. P. Collective bacterial dynamics revealed using a three-dimensional population-scale defocused particle tracking technique. Appl. Environ. Microbiol. 72, 4987–4994 (2006). 16820497 10.1128/AEM.00158-06 PMC1489374

[b22] Li G. & Tang J. Accumulation of microswimmers near a surface mediated by collision and rotational brownian motion. Phys. Rev. Lett. 103, 078101 (2009). 19792689 10.1103/PhysRevLett.103.078101 PMC2818302

[b23] Edwards M. R., Carlsen R. W., Zhuang J. & Sitti M. Swimming characterization of Serratia marcescens for bio-hybrid micro-robotics. J. Micro-Bio Robot. 9, 47–60 (2014).

[b24] Duffy K. & Ford R. Turn angle and run time distributions characterize swimming behavior for Pseudomonas putida. J. Bacteriol. 179, 1428–1430 (1997). 9023235 10.1128/jb.179.4.1428-1430.1997 PMC178849

[b25] Theves M., Taktikos J., Zaburdaev V., Stark H. & Beta C. A bacterial swimmer with two alternating speeds of propagation. Biophys. J. 105, 1915–1924 (2013). 24138867 10.1016/j.bpj.2013.08.047 PMC3797586

[b26] Berg H. & Brown D. Chemotaxis in *Escherichia coli* analysed by three-dimensional tracking. Antibiot. Chemother. 19, 55–78 (1974). 4618743 10.1159/000395424

[b27] Locsei J. T. Persistence of direction increases the drift velocity of run and tumble chemotaxis. J. Math. Biol. 55, 41–60 (2007). 17354016 10.1007/s00285-007-0080-z

[b28] Vladimirov N., Lebiedz D. & Sourjik V. Predicted auxiliary navigation mechanism of peritrichously flagellated chemotactic bacteria. PLoS Comput. Biol. 6, e1000717 (2010). 20333235 10.1371/journal.pcbi.1000717 PMC2841612

[b29] Taktikos J., Stark H. & Zaburdaev V. How the motility pattern of bacteria affects their dispersal and chemotaxis. PLoS ONE 8, e81936 (2013). 24391710 10.1371/journal.pone.0081936 PMC3876982

[b30] Turner L., Ryu W. & Berg H. Real-time imaging of fluorescent flagellar filaments. J. Bacteriol. 182, 2793–2801 (2000). 10781548 10.1128/jb.182.10.2793-2801.2000 PMC101988

[b31] Saragosti J., Silberzan P., Buguin A. & Modeling E. coli tumbles by rotational diffusion. Implications for chemotaxis. PLoS ONE 7, e35412 (2012). 22530021 10.1371/journal.pone.0035412 PMC3329434

[b32] Darnton N. C., Turner L., Rojevsky S. & Berg H. C. On torque and tumbling in swimming *Escherichia coli*. J. Bacteriol. 189, 1756–1764 (2007). 17189361 10.1128/JB.01501-06 PMC1855780

[b33] Altindal T., Xie L. & Wu X.-L. Implications of three-step swimming patterns in bacterial chemotaxis. Biophys. J. 100, 32–41 (2011). 21190654 10.1016/j.bpj.2010.11.029 PMC3010836

[b34] Sager B. M., Sekelsky J. J., Matsumura P. & Adler J. Use of a computer to assay motility in bacteria. Anal. Biochem. 173, 271–277 (1988). 3056105 10.1016/0003-2697(88)90189-3

[b35] Sandler O. . Lineage correlations of single cell division time as a probe of cell-cycle dynamics. Nature 519, 468–471 (2015). 25762143 10.1038/nature14318

[b36] Whitman W. B., Coleman D. C. & Wiebe W. J. Prokaryotes: the unseen majority. Proc. Natl Acad. Sci. USA 95, 6578–6583 (1998). 9618454 10.1073/pnas.95.12.6578 PMC33863

[b37] Terry K., Williams S. M., Connolly L. & Ottemann K. M. Chemotaxis plays multiple roles during Helicobacter pylori animal infection. Infect. Immun. 73, 803–811 (2005). 15664919 10.1128/IAI.73.2.803-811.2005 PMC547030

[b38] Lane M. C., Alteri C. J., Smith S. N. & Mobley H. L. T. Expression of flagella is coincident with uropathogenic *Escherichia coli* ascension to the upper urinary tract. Proc. Natl Acad. Sci. USA 104, 16669–16674 (2007). 17925449 10.1073/pnas.0607898104 PMC2034267

[b39] Mitchell J. G. & Kogure K. Bacterial motility: links to the environment and a driving force for microbial physics. FEMS Microbiol. Ecol. 55, 3–16 (2006). 16420610 10.1111/j.1574-6941.2005.00003.x

[b40] Berendsen R. L., Pieterse C. M. J. & Bakker P. A. H. M. The rhizosphere microbiome and plant health. Trends Plant Sci. 17, 478–486 (2012). 22564542 10.1016/j.tplants.2012.04.001

[b41] López-Garca S. L. . In-furrow inoculation and selection for higher motility enhances the efficacy of Bradyrhizobium japonicum nodulation. Agron. J. 101, 357–363 (2009).

[b42] Van Houdt R. & Michiels C. W. Biofilm formation and the food industry, a focus on the bacterial outer surface. J. Appl. Microbiol. 109, 1117–1131 (2010). 20522145 10.1111/j.1365-2672.2010.04756.x

