# Supplementary material for: High-throughput 3D tracking of bacteria on a standard phase contrast microscope
Source: Nat Commun. 2015 Nov 2;6:8776. doi: 10.1038/ncomms9776 (PMC4659942; doi:10.1038/ncomms9776)
Supplement: Supplementary Information — Supplementary Figures 1-6, Supplementary Discussion 1-3 and Supplementary References [file ncomms9776-s1.pdf]

Supplementary Figures

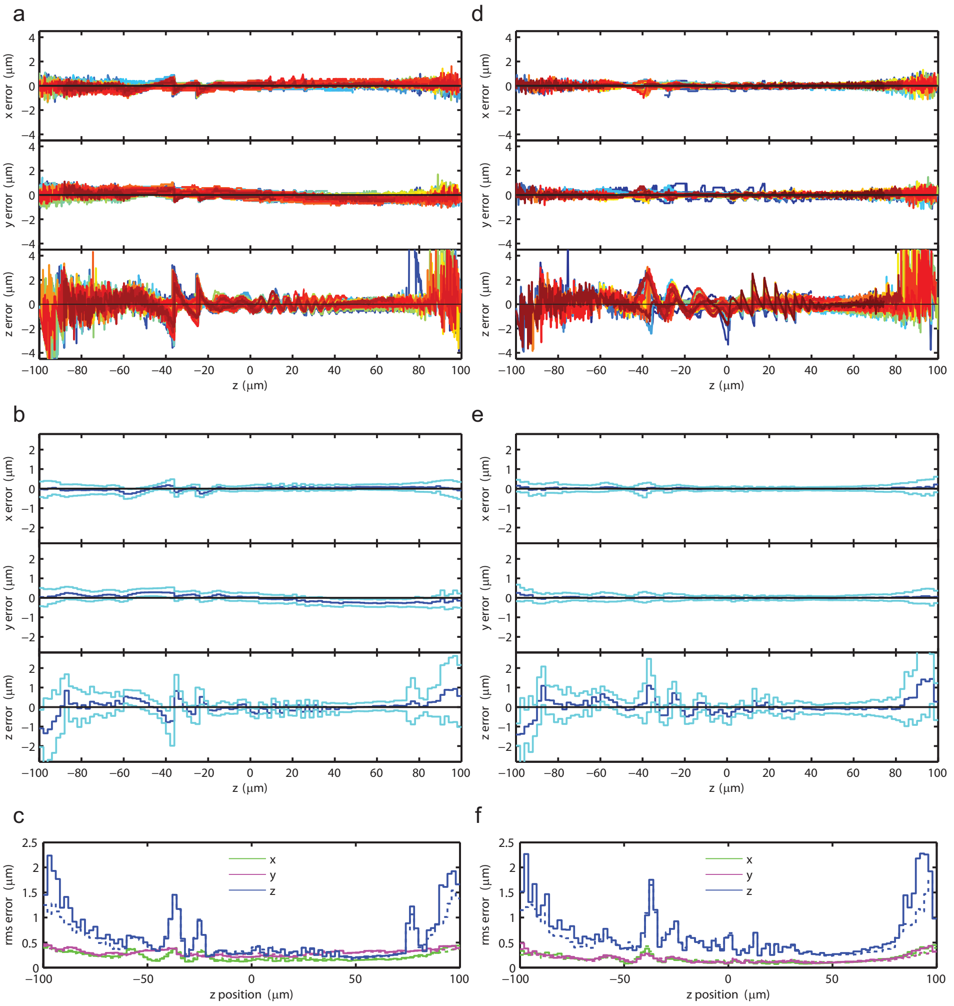

Supplementary Figure 1: Localization errors in 3D tracking of glass beads and bacteria against the reference stack shown in Fig. 2 of the main text. Error estimates were obtained for 73 1 μm glass beads (a-c) and for 48 *E. coli* bacteria (d-f). a,d) Raw errors plotted against z position, b,e) mean (dark blue) $\pm$ standard deviation (light blue) of localization errors for bins in z with a width of 2 μm. c,f) root mean square (rms) localization errors. The dashed line indicates the result obtained when the raw image data are binned 2x2 pixels laterally.

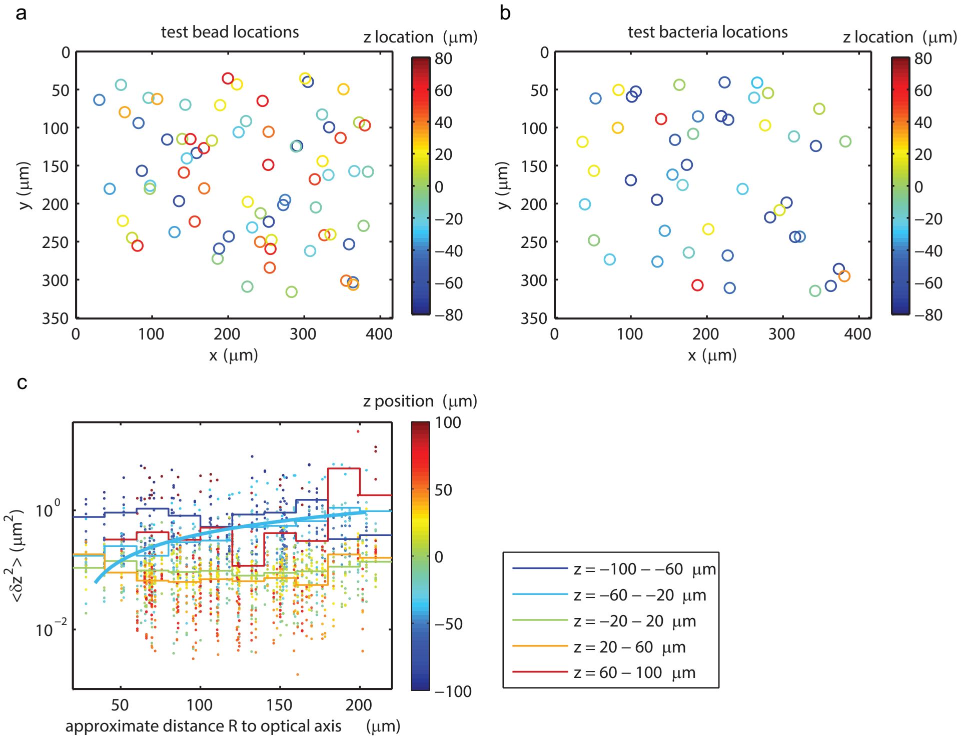

Supplementary Figure 2: Localization errors in z and object location. Localization errors were determined from tracking z stacks of immobilized, randomly distributed test objects, namely 73 beads (a) and 48 bacteria (b). The z location given by the colormap gives the z position of the object relative to the middle of the z stack. c) Dependence of mean square z position error on distance R from the optical axis. The underlying scatter plot shows squared z residuals averaged over $5\,\mu$m bins in z. The stair plots shown as solid lines are based on the same data, but averaged over $40\,\mu$m bins in z and $20\,\mu$m bins in R. Only for one range of z positions, a statistically significant correlation under a Student t-test ($p \approx 10^{-4}$), indicated by a bold solid line, is apparent.

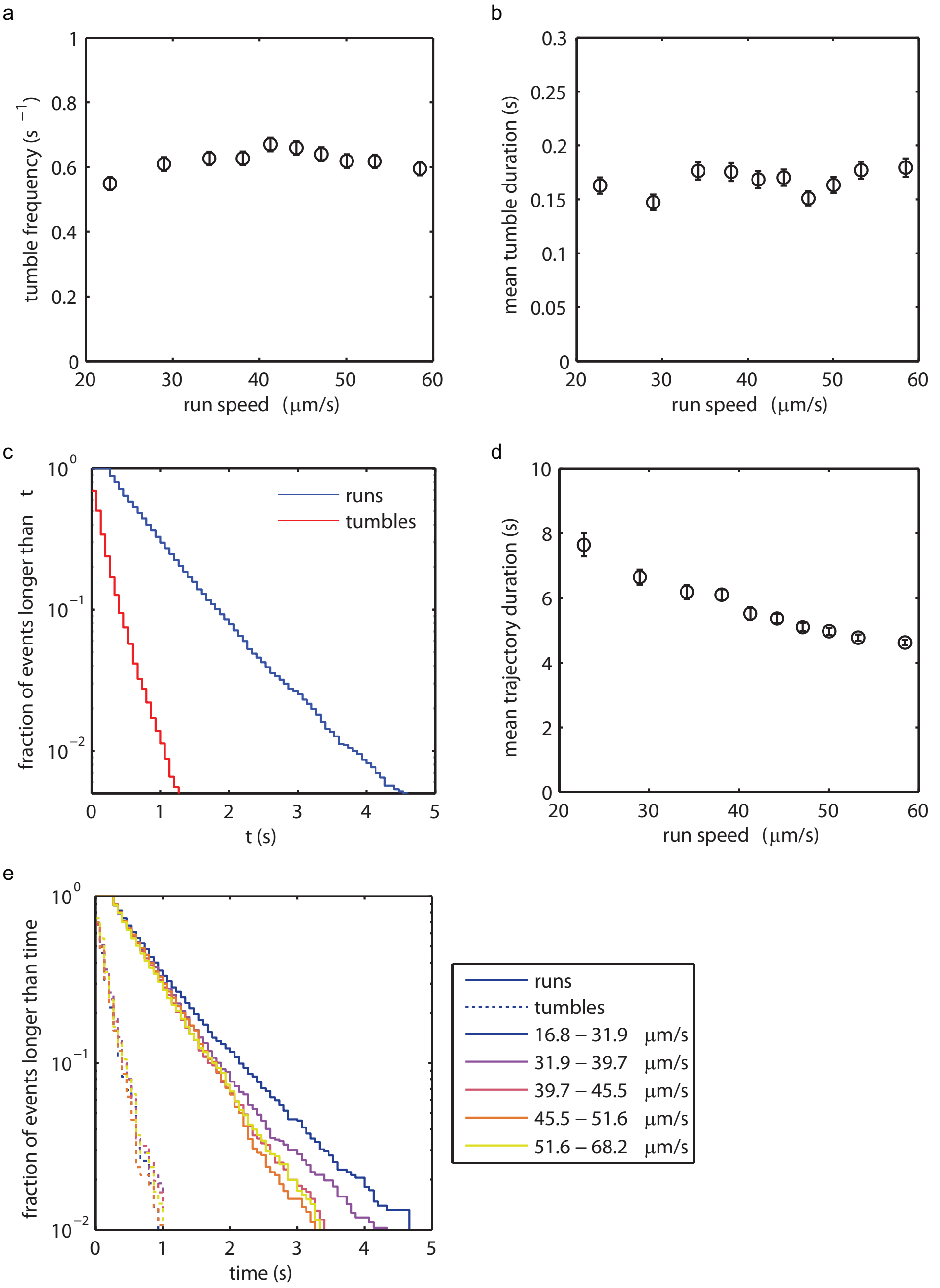

Supplementary Figure 3: Extended *E. coli* statistics. The analysis is based on 6 recordings of 100 s each from 4 different days. In 14 188 s of total trajectory time from 2551 motile bacteria with a minimum trajectory duration of 3 s, we identified 6015 runs and 8350 tumbles. All error bars represent standard errors of the mean. a) Average tumble frequency and b) average tumble duration plotted against run speed. c) 1− the cumulative distribution function for runs and tumbles, d) Average trajectory durations as a function of run speed for those trajectories on which the run-tumble analysis was performed (longer than 3 s). e) Run and tumble duration distributions for different run speeds. Run speed ranges in a,b,d,e are chosen so as to contain approximately equal total trajectory time.

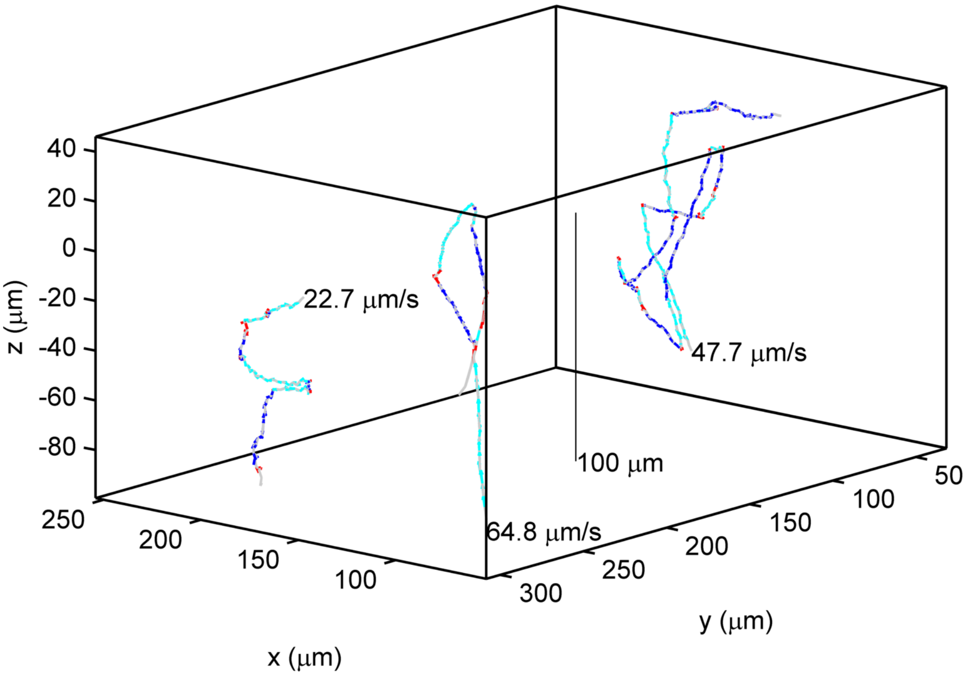

Supplementary Figure 4: Interactive 3D graphic of three example trajectories for *E. coli* bacteria. Median run speeds from left to right: $22.7\,\mu\mathrm{m\,s^{-1}}$, $64.8\,\mu\mathrm{m\,s^{-1}}$, $47.7\,\mu\mathrm{m\,s^{-1}}$. Scale bar: $100\,\mu$m.

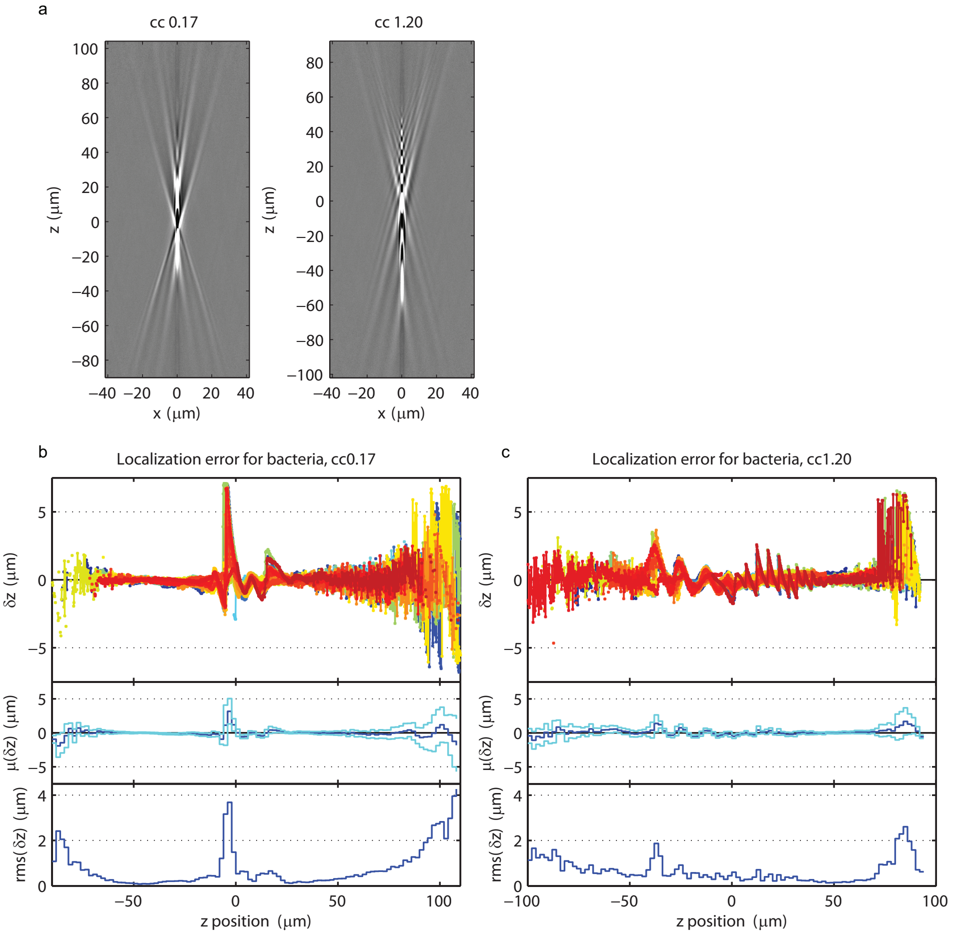

Supplementary Figure 5: Effect of deliberately introduced optical aberrations on localization errors. a) Vertical slice through reference stacks obtained in the absence and presence of deliberately introduced spherical aberrations, corresponding to correction collar settings of 0.17 mm and 1.2 mm, respectively. Both reference stacks were obtained by averaging image stacks obtained from an identical set of 42 glass beads. Note that the reference stack used in Fig. 1 is based on a larger number of contributing stacks and is therefore not identical to the one shown here on the right hand side. Absolute z positions were assigned such that $z = 0$ for the position with the highest contrast, defined here as an image's maximum intensity deviation from the background. b,c) Localization errors in z in the absence (b) and presence (c) of deliberately introduced spherical aberrations. The data were obtained for image stacks of an identical set of 34 bacteria localized against the reference stacks shown in a.

Supplementary Figure 6: Run length sampling bias in 2D slicing and 3D tracking. a) Schematic of definitions used in Supplementary Discussion 1. b) Run length sampling efficiency in 2D slicing according to Eq. 17. c) Errors for exponential run length distribution in 2D slicing. Black, left axis: Ratio of measured to true average run length according to Eq. 21. Gray, right axis: Overall sampling efficiency according to Eq. 23. d) Histogram of trajectory durations obtained for 3D tracking of *E. coli.* The minimum duration cutoff of 3 s used in the run-tumble data analysis is marked by a dashed line. e) Run duration sampling bias in 3D tracking. Solid line: computed on the basis of the full trajectory duration distribution in panel c, according to Eq. 28. Dashed line: Same, but with truncated trajectory duration distribution ($T \geq 3\,\mathrm{s}$.) Dotted line: Run duration sampling efficiency for 2D slicing, assuming a constant swimming speed of $v = 30\,\mu\mathrm{m\,s}^{-1}$ and a slice thickness of $d = 6\,\mu\mathrm{m}$. f) Errors for exponential run duration distribution in 3D tracking, based on sampling efficiencies plotted in panel e. Black, left axis: Ratio of measured to true average run duration. Gray, right axis: Overall sampling efficiency. Solid, dashed, and dotted lines: as in panel e.

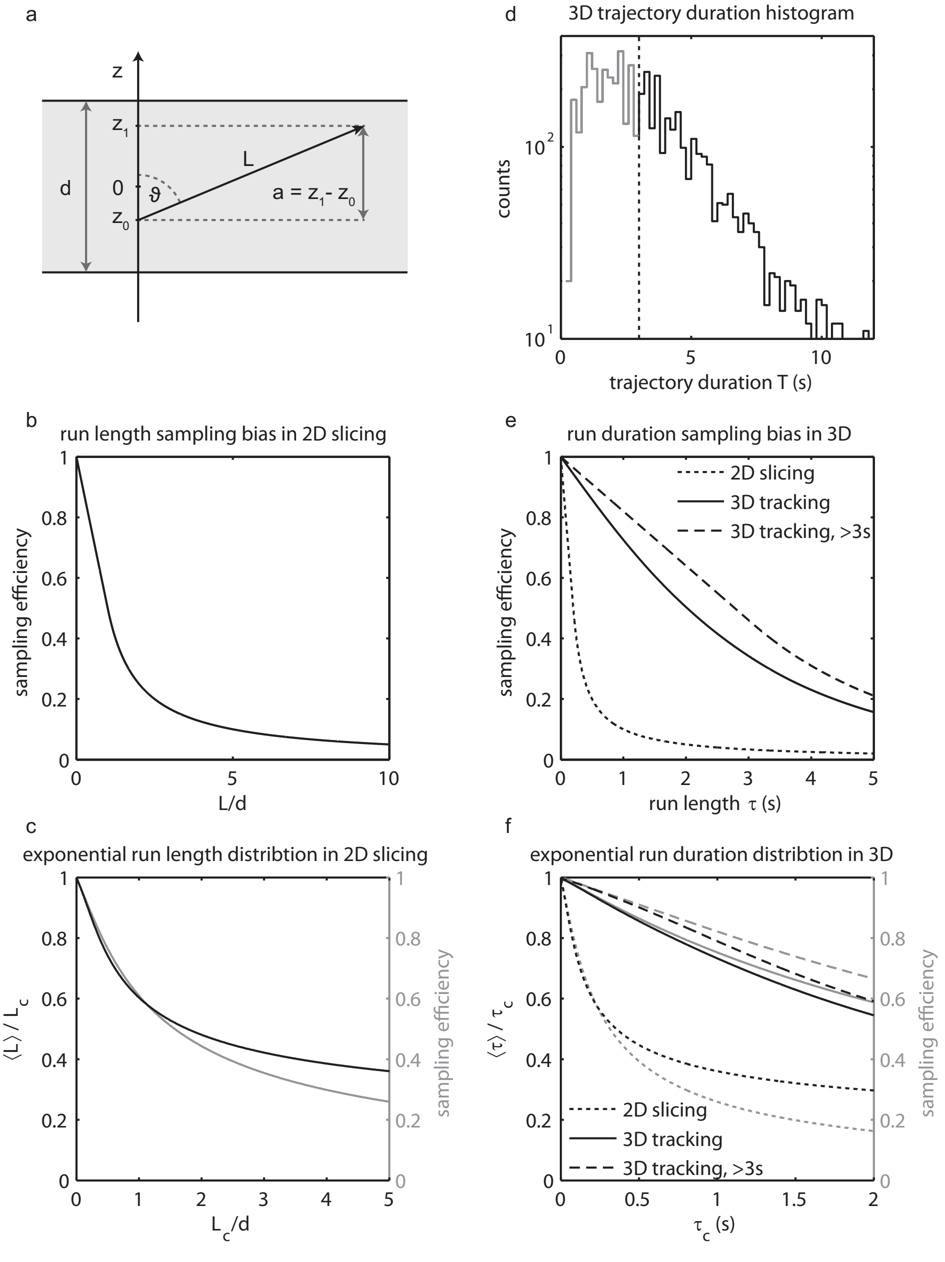
a
z
z_1
L
d
0
ϑ
$a = z_1 - z_0$
z_0
d
3D trajectory duration histogram
counts
trajectory duration T (s)
b
run length sampling bias in 2D slicing
sampling efficiency
L/d
e
run duration sampling bias in 3D
2D slicing
3D tracking
3D tracking, >3s
sampling efficiency
run length τ (s)
c
exponential run length distribtion in 2D slicing
⟨L⟩ / L_c
sampling efficiency
L_c/d
f
exponential run duration distribtion in 3D
⟨τ⟩ / $τ_c$
sampling efficiency
2D slicing
3D tracking
3D tracking, >3s
$τ_c$ (s)

Supplementary Discussion 1: Measurement and sampling considerations for 2D and 3D tracking

Speed measurement errors in 2D

In 2D projection

In 2D projection microscopy, objects moving in 3D (x,y,z) are observed in the (x,y)-plane, that is, the z coordinate is omitted. Here, we determine the root mean square error in speed measurements that arise in projection.
The 3D velocity of an object can be written as

$$\mathbf{v} = \begin{pmatrix} v\cos\phi\sin\vartheta \\ v\sin\phi\sin\vartheta \\ v\cos\vartheta \end{pmatrix}, \tag{1}$$

where v is the speed, i.e. the absolute magnitude of the velocity, $\vartheta \in [0, \pi]$ refers to the angle between $\mathbf{v}$ and the positive z axis, and $\phi \in [0, 2\pi]$ is the angle between $\mathbf{v}$'s projection into the x-y plane and the positive x axis. The speed in 2D projection, v_p, is then smaller than the true speed v:

$$v_\mathrm{p} = v\sin\vartheta\,. \tag{2}$$

The resulting relative error in speed measurement r is stgiven by

$$r = \frac{\delta v}{v} = \frac{v - v_\mathrm{p}}{v} = 1 - \sin\vartheta. \tag{3}$$

Assuming that all directions of movement are equally likely in 3D, the angle ϑ is distributed according to

$$g(\vartheta) = \frac{dp}{d\vartheta} = \frac{1}{2}\sin\vartheta \quad \text{for} \quad \vartheta \in [0, \pi]\,, \tag{4}$$

where p refers to probability. The mean square relative error for the speed measurement is then

$$\mathrm{rms}(r)^2 = \int_0^\pi d\vartheta\,(1 - \sin\vartheta)^2 g(\vartheta) = \frac{5}{3} - \frac{\pi}{2}. \tag{5}$$

Hence the root mean square error is given by

$$\mathrm{rms}(r) = \sqrt{\frac{5}{3} - \frac{\pi}{2}} \approx 0.31. \tag{6}$$

In 2D slicing

Eq. 3 shows that speed measurement errors are smallest when the observed object moves in plane, that is, at an angle ϑ that is close to $\pi/2$. This can be achieved by 2D slicing, that is, limiting observations to a thin z slice of thickness d, usually determined by the depth of field of the optical setup. We

assume that an observed bacterium is performing straight runs of length L and arbitrary orientation in 3D, and that measurements are evaluated only for runs that lie fully within the slice (see Supplementary Fig. 6a).
In practice, typical depths of field d for standard medium magnification microscopy objective lenses are several μm, whereas typical bacterial run lengths are a few tens of μm, so that usually $L > d$. Hence only runs at angles $\vartheta \in [\frac{\pi}{2} - \vartheta_{\max}, \frac{\pi}{2} + \vartheta_{\max}]$ are detected, where ϑ is defined as above and $\cos\vartheta_{\max} = d/L$. Then, according to Eq. 3, the maximal relative error in speed measurement given by

$$r_{\max} = 1 - \sin\vartheta_{\max} = 1 - \sqrt{1 - \left(\frac{d}{L}\right)^2} \, . \tag{7}$$

Using typical values for *E. coli* of a swimming speed $v = 30\,\mu\mathrm{m\,s^{-1}}$ and a run duration $\tau = 1\,\mathrm{s}$ together with a typical depth of field of $d = 6\,\mu$m, we obtain $L/d = v\tau/d = 5$, resulting in $r_{\max} \approx 0.02$. So errors in speed measurements can be greatly reduced by performing 2D slicing rather than 2D projection.

Turning angle measurement errors and biases in 2D

Turning angle measurement errors in 2D projection

When a turn by an angle θ is projected into a two-dimensional plane, the angle α observed in plane can have any value from 0 to 180° degrees, depending on the orientation of the incoming and outgoing runs. Here we compute the root mean square measurement error in turning angle that arises as a result.
We assume that the orientation of the incoming run is distributed isotropically, and that the orientation of the outgoing run is distributed rotationally symmetrically about the axis of the incoming run but constrained to create an angle θ with it. For this situation, Ref. [1] derives the first two moments of the distribution of the projected angle α:

$$\langle\alpha\rangle = \theta \, , \tag{8}$$

$$\langle\alpha^2\rangle = 4\left(1 - \frac{\theta}{\tan\theta}\right) + 2\pi\left(\frac{\theta}{2} - \tan\frac{\theta}{2}\right) . \tag{9}$$

We are interested in the root mean square error in angle measurement, $\mathrm{rms}(\delta\alpha) = \mathrm{rms}(\alpha - \theta)$:

$$\mathrm{rms}(\delta\alpha)^2 = \left\langle(\alpha - \theta)^2\right\rangle = \langle\alpha^2\rangle - \theta^2 \tag{10}$$

$$= 4\left(1 - \frac{\theta}{\tan\theta}\right) + 2\pi\left(\frac{\theta}{2} - \tan\frac{\theta}{2}\right) - \theta^2 \, , \tag{11}$$

where we have used Eq. 8 and Eq. 9. We plot $\mathrm{rms}(\delta\alpha)$ in Figure 1 of the main text.

In situations where only the mean turning angle $\langle\theta\rangle$ is of interest, one may exploit that the mean projected turning angle $\langle\alpha\rangle$ matches $\langle\theta\rangle$ as follows from Eq. 8. For the purpose of bacterial motility, however, the relevant parameter for the prediction of key features such as the effective diffusion coefficient and the chemotactic drift velocity is not $\langle\theta\rangle$ but the persistence $\langle\cos\theta\rangle$ [2, 3]. In contrast to $\langle\theta\rangle$, the persistence is not always conserved in projection. In special cases where $\langle\cos\theta\rangle = \cos\langle\theta\rangle$ it can be obtained as $\langle\cos\theta\rangle = \cos\langle\alpha\rangle$. These cases include delta-distributed turning angles and distributions that are symmetric about 90°, but not for instance the asymmetric broad turning angle distribution of *E. coli.*

In addition, Eq. 8 is only applicable if no sampling bias exists, that is, all turning events are equally likely to be detected, regardless of orientation and turning angle. As tumble detection is usually based on observed velocity changes, the incomplete velocity information obtained in 2D projection renders the avoidance of such sampling biases challenging.

Furthermore, the turning angle measurement error arising from Eq. 9 decreases the precision to which average turning angles can be determined in 2D projection, and thereby hampers the detection of statistically significant differences between individuals. According to the central limit theorem, the standard error of the mean, $\sigma(\langle x\rangle)$, for a sample of N independent measurement values x drawn from a distribution with variance $\sigma^2(x)$ and mean x_0 is given by

$$\sigma^2(\langle x\rangle) = \frac{\sigma^2(x)}{N}\,. \tag{12}$$

Consequently, the variance $\sigma^2(x)$ determines how many measurements N are required to determine x_0 with a given precision $\sigma(\langle x\rangle)$. Assuming an underlying turning angle distribution with variance $\sigma^2(\theta)$ and mean θ_0, the variance of the turning angle measured in projection is given by the sum of intrinsic variance $\sigma^2(\theta)$ and average measurement error variance and can be approximated by

$$\sigma^2(\alpha) = \sigma^2(\theta) + \sigma^2(\alpha)|_{\theta_0} = \sigma^2(\theta) + \mathrm{rms}(\delta\alpha)^2|_{\theta_0}\,, \tag{13}$$

where we assume that the measurement error does not vary appreciably over the range covered by the true turning angle distribution. In the example of *V. alginolyticus* flick angles, we observe typical variances of $\sigma^2(\theta) \approx (15°)^2$ for individuals. For the flick angle range of $55-125°$, Eq. 11 yields measurement errors of at least 21°. Consequently, the presence of projection errors would increase the number of data points that are required for determining θ_0 to a given precision by a factor of at least $(21^2 + 15^2)/15^2 \approx 3$ in this application.

Turning angle sampling bias in 2D slicing

The above measurement errors are smallest for turns that lie fully in plane, and hence can be minimized by demanding that both runs flanking the turn

lie fully within a thin slice. As the majority of turns occurring within the slice however are not in-plane, the sampling efficiency is low. Estimating the sampling efficiency in 2D slicing analytically is challenging, therefore we evaluate the problem numerically.

We assume that i) tumbles occur with equal probability at all positions within a slice of thickness d, ii) the orientation of the incoming run is distributed isotropically, iii) the orientation of the outgoing run is distributed rotationally symmetrically about the axis of the incoming run but constrained to create an angle θ with it, iv) both the incoming and the outgoing run have length L and v) must lie fully within the slice. In practice, we first create events according to assumptions (i-iv) and then determine the sampling efficiency as the fraction of events that are rejected by assumption (v). In Figure 1 of the main text, we plot the result obtained for $L/d = 5$. As the sampling efficiency varies as a function of the turning angle θ, a bias in sampling results, and the measured angle distribution does not match the true one.

Run length sampling bias in different tracking methods

Run length sampling bias in 2D slicing

Bias as a function of run length

In 2D slicing, all events within a slice of thickness d are observed. The run length sampling efficiency R_{2D} is given by the fraction of runs starting inside the slice that also end inside the slice. As 2D slicing imposes a geometric constraint on detection, it is convenient to describe runs by their spatial length L rather than their temporal duration $\tau = L/v$ where v is the run speed. If the sampling efficiency is not constant as a function of run length L, a detection bias arises, and the measured distribution of run lengths does not match the true distribution.

To estimate the run length sampling efficiency in 2D slicing, we make the following simplifying assumptions: i) Runs can start at any position within the slice with equal probability, ii) runs are straight, and iii) all run orientations are equally likely. We denote the run starting position by z_0. According to assumption (i), its position is distributed according to

$$f(z_0) = \frac{dp}{dz_0} = \begin{cases} 1/d, & \text{if } |z_0| \leq d/2 \\ 0, & \text{otherwise.} \end{cases} \tag{14}$$

The run of length L ends at $z_1 = z_0 + a$ where, according to assumption (ii), $a = L\cos\vartheta$ (see Supplementary Fig. 6a). Following assumption (iii), ϑ is distributed according to Eq. 4, which we use to obtain the probability distribution for a:

$$h(a) = \frac{dp}{da} = g(\vartheta)\left|\frac{d\vartheta}{a}\right| = \begin{cases} \frac{1}{2L}, & \text{if } |a| \leq L \\ 0, & \text{otherwise.} \end{cases} \tag{15}$$

We are interested in the probability of z_1 falling inside the range $[-d/2, d/2]$:

$$R_{2\mathrm{D}} = p(|z_1| \leq d/2) = \int_{-d/2}^{d/2} dz_1 \int_{-d/2}^{d/2} dz_0 \, f(z_0) h(z_1 - z_0) \, . \tag{16}$$

Solving the integral yields

$$R_{2\mathrm{D}}(L/d) = \begin{cases} 1 - \frac{L}{2d}, & \text{if } L \leq d \\ \frac{d}{2L}, & \text{if } L > d, \end{cases} \tag{17}$$

which is plotted in Supplementary Fig. 6b for a range L/d that covers values typical for bacterial motility experiments. Given typical depths of field d for standard medium magnification microscopy objective lenses on the order of several μm, typical swimming speeds v in the range of 10s of $\mu\mathrm{m}\,\mathrm{s}^{-1}$, and typical run durations τ of hundreds of ms, we obtain a typical range of $L/d = v\tau/d \approx 1 - 10$, which is covered in Supplementary Fig. 6b.
If there is a distribution of run speeds $U(v)$ within the population as is commonly observed experimentally, and one would like to obtain the sampling efficiency as a function of run duration τ rather than run length L, it can be obtained in the form of a weighted average of Eq. 17:

$$\widetilde{R}_{2\mathrm{D}}(\tau) = \int_0^\infty dv \, R_{2\mathrm{D}} \left(\frac{v\tau}{d} \right) U(v) \, . \tag{18}$$

Overall sampling bias for exponential run length distribution
Oftentimes, run durations τ have approximately exponential distributions. If we neglect variability in bacterial swimming speeds, then also the run lengths $L = vt$ will be exponentially distributed,

$$r(L) = \frac{dp}{dL} = \frac{e^{-L/L_\mathrm{c}}}{L_\mathrm{c}}, \tag{19}$$

with some characteristic length L_c. Given the sampling bias as a function of run length given by Eq. 17, one may ask what fraction F of runs is detected, given L_c and d:

$$F = \int_0^\infty dL \, r(L) R_{2\mathrm{D}}(L/d). \tag{20}$$

The integration yields

$$F(L_\mathrm{c}/d) = 1 + \frac{1}{2} \left\{ -\frac{L_\mathrm{c}}{d} + e^{-d/L_\mathrm{c}} \left(\frac{L_\mathrm{c}}{d} - 1 \right) + \frac{d}{L_\mathrm{c}} E(d/L_\mathrm{c}) \right\}, \tag{21}$$

where $E(x)$ is the exponential integral function:

$$E(x) := \int_x^\infty dt \, \frac{e^{-t}}{t}. \tag{22}$$

Eq. 21 is plotted in Supplementary Fig. 6c.

Resulting error in average run length
Often, the average run length is of interest. In case of an exponential run length distribution, the true average coincides with the characteristic length L_{c}. Run length sampling biases may however lead to discrepancies between the measured and true average. The measured average length is given by

$$\langle L \rangle = \frac{\int_0^\infty dL\, L\, r(L)\, R_{2\mathrm{D}}(L/d)}{\int_0^\infty dL\, r(L)\, R_{2\mathrm{D}}(L/d)} \tag{23}$$

$$= \frac{A}{F(L_{\mathrm{c}}/d)}. \tag{24}$$

The integral in the numerator of Eq. 25 yields

$$A = L_{\mathrm{c}} \left\{ 1 - \frac{L_{\mathrm{c}}}{d} \left(1 - e^{-d/L_{\mathrm{c}}} \right) \right\}. \tag{25}$$

Because the sampling efficiency $R(L/d)$ decays with increasing L, $\langle L \rangle < L_{\mathrm{c}}$. We plot the ratio $\langle L \rangle / L_{\mathrm{c}}$ in Supplementary Fig. 6c.

Run duration bias in arbitrary tracking methods

Run duration detection biases are present in any tracking method, regardless of dimensionality, as a result of the finite duration of trajectories. The following considerations hence are completely generic. We only opted to examine 2D slicing separately because, in that specific case, geometric considerations present a convenient route for analysis that does not require knowledge of trajectory duration distributions.

Detection bias a function of run duration
Given a trajectory of duration T and a run of duration τ that begins in it, the probability for the run to end before the trajectory ends is $\frac{T-\tau}{T}$ if $\tau < T$ and 0 otherwise. Hence the sampling efficiency is given by

$$B(\tau/T) = \begin{cases} \frac{T-\tau}{T}, & \text{if } \tau \leq T \\ 0, & \text{if } \tau > T, \end{cases} \tag{26}$$

Experimentally, one generally obtains a distribution $S(T)$ of trajectory durations. Under the simplifying assumption that there is no correlation between a trajectory's duration T and the duration of the runs τ in it - that is, the tumble frequency does not affect the average length of trajectories - , the resulting run duration sampling bias may be computed as

$$\widetilde{R}_{3\mathrm{D}}(\tau) = \frac{\int_0^\infty dT\, B(\tau/T)\, T\, S(T)}{\int_0^\infty dT\, T\, S(T)}, \tag{27}$$

which takes into account that trajectories on average contribute a number of runs proportional to their duration. The denominator corresponds to the average trajectory duration. Inserting Eq. 26 into the numerator yields

$$\widetilde{R}_{3\mathrm{D}}(\tau) = \frac{(\langle T \rangle_{T \geq \tau} - \tau)\, p(T \geq \tau)}{\langle T \rangle}. \tag{28}$$

If the distribution $S(T)$ of trajectory durations is known, the resulting bias $\widetilde{R}_{3\mathrm{D}}(\tau)$ can be readily computed.

Run duration bias in our 3D tracking
Supplementary Fig. 6d shows a histogram of trajectory durations for the dataset we used for *E. coli* run-tumble analysis. In panel e of the same figure we plot the numerically computed run duration sampling bias, both with the full and the truncated distribution of trajectory durations used in the run-tumble analysis. For comparison, we also show the sampling bias for 2D slicing using $L/d = v\tau/d$ with the assumption of $v/d = 30\,\mu\mathrm{m\,s}^{-1}/\,6\,\mu\mathrm{m} = 5\,\mathrm{s}^{-1}$.
We note that the validity of the simplifying assumption of no correlation between tumble frequency and trajectory duration depends on the nature of the causes for trajectory termination. If exiting the tracking range constitutes the major cause of termination, the assumption is not valid because the average time it takes a bacterium to cross a given distance depends on the average run duration [4]. Numerical simulations could be employed to more rigorously evaluate the sampling bias incurred in this situation. At higher bacterial densities, however, diffraction ring overlaps constitute the major cause of trajectory termination, and the simplification may be valid.

Error in average run duration
Analogous to the 2D slicing case, it is of interest to determine the error in the measured average run duration, under the assumption of an underlying exponential distribution $\widetilde{r}(\tau)$ with average τ_c. The computation is the temporal equivalent of Eq. 23:

$$\langle\tau\rangle = \frac{\int_0^\infty d\tau\,\tau\,\widetilde{r}(\tau)\,\widetilde{R}_{3\mathrm{D}}(\tau)}{\int_0^\infty d\tau\,\widetilde{r}(\tau)\,\widetilde{R}_{3\mathrm{D}}(\tau)}\,. \tag{29}$$

For our 3D tracking method, we plot the numerically obtained result in Supplementary Fig. 6f. We note that for realistic *E. coli* run durations of 0.5-1 s, our 3D tracking method is expected to yield a mean run duration that is 10-20% too small, while 2D slicing with the same parameters as above is off by more than a factor of 2.

Speeds, trajectory durations, and statistics

Faster bacteria are more likely, per unit time, to meet the conditions for trajectory termination such as exiting the tracking range. Similarly, they are also more likely, per unit time, to newly enter the tracking volume. As a result, trajectories are generally biased to be of shorter duration, yet more numerous, for faster individuals. Therefore a selection bias in bacterial swimming speed may be incurred by limiting analyses to trajectories with a given minimum duration. In our *E. coli* study, however, requiring a minimum trajectory duration of 3 s only decreases the population average of the

speed by less than 3% from 40.2 μm s^{-1} to 39.0 μm s^{-1} for motile bacteria (having a median trajectory speed larger than 17 μm s^{-1}). In computing these population averages, trajectories were weighted by their duration. In contrast to trajectory number, total trajectory time obtained for bacteria of a given speed range is an unbiased measure of the number of these bacteria in the population.

Supplementary Figure 3d shows the average trajectory duration as a function of speed for the trajectories used in our *E. coli* run-tumble analysis (all trajectories with a median speed larger than 17 μm s^{-1} and a minimum duration longer than 3 s). The average trajectory duration changes by nearly a factor of two over the speed range of 20-60 μm s^{-1}. Consequently, the severity of run length sampling biases varies with speed. This is apparent in the increased weight at long times in the run length distributions of slower bacteria compared to those of faster ones (Supplementary Figure 3e). The presence of sampling bias could explain why our overall run duration distribution (Fig. 3c) does not show non-exponential long-time tails as pronounced as have been observed in physical tracking [5] where average trajectory durations were on the order of tens of seconds. At the other extreme, these tails would likely not be observable at all in 2D slicing, given the severity of run length sampling biases incurred by this method (Fig. 6b).

Supplementary Discussion 2: Additional notes on performance.

Data and computation

Our tracking method exploits recent advances in the availability and affordability of large fast detectors, digital storage capacity, and computing power that have rendered the data acquisition and analysis practical with standard hardware. In its current implementation, 100 s of recordings at 15 Hz occupy $\sim$ 15 GB of storage space and, depending on bacterial density, take 5-20 h to process on a high-end consumer desktop computer (Intel i7 870 quad core 2.93 GHz processor, 16 GB RAM). Binning the data by a factor of 2 in each lateral direction, however, we find actually slightly improves the spatial resolution (Supplementary Fig. 1), and results in a decrease by a factor of $\sim$ 4 in storage requirements and processing time. Increased computational efficiency may yet be achieved by further optimization of data processing. In particular, the current implementation, while efficient at tracking known bacteria from frame to frame, could be accelerated significantly by an improved procedure for initializing trajectories for new bacteria entering the tracking volume.

Spatial resolution

Sources of localization errors

The z resolution of our technique is dictated by the variation of the observed diffraction pattern with z position. As a general rule, errors are smaller where the pattern varies more quickly with distance, e.g. in the range of 0 - 80 μm. Larger errors arise where the pattern varies more slowly with z position, e.g. in the range of -40 to -20 μm. It is expected that the achievable localization precision via cross-correlations is smaller where the cross-correlation of neighbouring reference images decays more rapidly with neighbour distance.

In our application, we find that the coefficient of variation (defined as the standard deviation divided by the mean) of the mean square z localization error for 1 μm glass beads across the tracking range is $CV_{\rm z} \approx 1.65$. Because our error determination procedure combines data from many test beads at each z position, variations due to other error sources such as differences between beads are averaged out. We therefore interpret this number to reflect only the variation in error magnitude due to z.

By contrast, comparing the mean square z localization error between different glass beads, but at the same z position, we obtain an average coefficient of variation of $CV_{\rm b} \approx 1$, implying that differences between test objects are less significant to the magnitude of the z localization error than z position. Such differences between test objects include distance R to the optical axis, background intensity distribution and depth location in the sample. As test objects are randomly distributed in the sample volume (Supplementary

Fig. 2a,b), we expect that the distribution of distances R from the optical axis that they sample matches the one sampled in bacterial motility experiments. To test for an effect of R on the magnitude of the z localization errors, we bin mean square z localization errors by R and z (Supplementary Fig. 2c). Different z position ranges need to be considered separately because of the strong z-dependence of the error magnitude, and binning in R is necessary to average out differences other than R between test objects. We found a statistically significant correlation (probability of no correlation under a Student t-test $p \approx 10^{-4}$, while $p > 0.05$ elsewhere) between the mean square z localization error and R only for the z range of -60 to -20 μm (solid line in Supplementary Fig. 2c). Here the correlation coefficient is larger than 0.9, and the coefficient of variation of $CV_{\mathrm{R}} \approx 0.56$ sets an upper limit on the contribution of R to the localization error. As this value is significantly smaller than $CV_{\mathrm{z}} = 1.65$ and $CV_{\mathrm{b}} \approx 1$, we conclude that while the distance R to the optical axis is a factor affecting z localization precision, it is not the dominant one.

Furthermore, the average mean square z localization error for *E. coli* bacteria is only 17% larger than for spherical glass beads (see panels c and f in Supplementary Fig. 1), implying that asymmetry in the range of typical bacterial populations does not have a large effect on localization precision.

Practical implications

The micron-scale resolution of our technique is smaller than the typical size of most bacteria. It is sufficient for the analyses presented here as the typical displacement between frames due to swimming is larger than the typical localization error. For slower swimmers, acquisition frequencies may be decreased as long as the features of interest, e.g. runs, are still well sampled temporally. It seems likely that bacteria generally do not actively modulate their motility on time scales over which their displacement is smaller than their body size. For problems that require a higher spatial resolution, tracking may be restricted to a subset of the tracking range. For a range of 100 μm in z, the z localization error is only $\sim 0.5\,\mu$m. In addition, we find that further improvements in resolution over a smaller range can be achieved if no spherical aberrations are introduced deliberately (Supplementary Fig. 5). For instance, over a range of 40 μm in z, a z error smaller than ≤ 250 nm is possible. In this case, however, larger localization errors are present near the focus.

Temporal resolution

The temporal resolution of our technique is only limited by the detector readout speed (100 Hz full frame in our case). As phase contrast microscopy is typically not photon-limited, integration times can easily be decreased if illumination is increased to maintain the signal-to-noise ratio.

For any detection technique, motion blur can affect measured velocities if the

exposure time is similar to the time between exposures. In our application, the 5 ms exposure time used is significantly shorter than the time between exposures (67 ms) so that our speed measurements are unlikely to be affected by motion blur.

Furthermore, localization errors in z may be expected to arise from the fact that the measured diffraction pattern represents the time-averaged diffraction pattern during the exposure. A typical *E. coli* bacterium swimming at a speed of $40\,\mu\mathrm{m\,s^{-1}}$, however, traverses a distance of only $0.2\,\mu$m during the 5 ms exposure time. Across such small distances in z, the diffraction pattern barely changes, and hence the measured pattern is quite similar to the one that would have been obtained in the absence of motion blur, that is, in an infinitely short exposure. In any case, an upper bound for the localization error caused by motion blur is given by the distance traversed during the exposure, which is smaller than the spatial resolution our technique and significantly smaller than the body size of the bacterium. We therefore consider motion blur negligible in our application.

Applicability

The technique's applicability is not limited by refractive index variability in the range typically covered by bacteria, as illustrated by the fact that $1\,\mu$m silica beads, with a refractive index $n_{\mathrm{Si}} \approx 1.45$ higher than $n_{\mathrm{Ec}} \approx 1.39$ for *E. coli* [6], produce similar intensity patterns and can be used as a reference. Localization errors increase with increasing aspect ratio of the tracked object, but, on average, we find only slightly larger errors for *E. coli* bacteria than for silica beads (Supplementary Fig. 1). For significantly longer bacteria, deconvolution of the observed diffraction patterns with reference library images could be used to retrieve the bacterial shape and obtain a more accurate position of its centroid.

Comparison to Digital Holographic Microscopy

We limit our comparison to Digital Holographic Microscopy (DHM) as this is the only existing 3D bacterial tracking method that delivers a throughput, range, and resolution comparable to our technique. While DHM is a powerful method for 3D imaging, its implementation involves significant challenges with respect to hardware, computational efficiency, and storage requirements.

Typically, dedicated illumination sources such as lasers are used [7, 8], and special coatings on the sample chambers can be necessary to avoid problems arising from reflections [7]. In contrast, our technique requires no additional hardware beyond a phase contrast microscope and a camera, and the sample preparation is basic.

Furthermore, background correction in DHM can be cumbersome. For instance, Molaei et al. [7] record more than 20 times more images for background correction than for motility analysis. In contrast, the background

correction for our method requires no additional data.
While datasets recorded for DHM are 2D images that are similar in size and number to those acquired in our tracking technique, DHM requires a subsequent computational reconstruction of 3D intensity distributions, one z slice at a time. This reconstruction is computationally intense - Sheng et al. [9] report a processing time of 1 week for a recording 4 times smaller than the ones we typically process overnight, or, with recent improvements, within a few hours.
In addition, because each z slice in the reconstruction is as large the original 2D image, the reconstructed 3D dataset is necessarily significantly larger than the original recording. The number of slices can be reduced only at the expense of z range or resolution. For a z slice spacing of $0.5\,\mu$m over $200\,\mu$m as was used for *E. coli* [10], the 3D reconstruction is 400 times larger than the original data set. With typical recording sizes on the order of Gigabytes, the 3D reconstruction of a movie occupies approximately a Terrabyte. DHM thus comes with storage requirements that are at least transiently two to three orders of magnitude larger than our simple method and hence not easily compatible anymore with data storage hardware available on the consumer market.
From the 3D intensity distributions reconstructed in DHM, objects need to be identified and localized for each time point. Determining which positions in consecutive frames belong to the same trajectory can be computationally complex [11], but is rendered unnecessary in our method by a local search strategy for tracking each bacterium from one frame to the next.
Our simple technique in its present implementation already achieves the same tracking range as DHM [7], and, across half of the z range, also matches its $0.5\,\mu$m z resolution [7]. In addition, we demonstrate that achieved resolution is sufficient for retrieving key motility parameters. In summary, the current implementation of our 3D tracking method already provides a performance close to the best competitor technique, but at a greatly reduced level of technical difficulty.

Supplementary Discussion 3: *E. coli* swimming speed.

Bacterial swimming speeds can differ severely depending on growth conditions, growth phase, physical handling, and swimming medium. Consequently, swimming speeds reported for *E. coli* strain AW405, which we use here, vary substantially.

Berg & Brown [5] report swimming speeds for AW405 much lower than those we report here ($\sim 14\,\mu\mathrm{m\,s^{-1}}$ versus $\sim 40\,\mu\mathrm{m\,s^{-1}}$), but for bacteria grown in minimal medium with glycerol as the only carbon source. We grow AW405 in tryptone broth, a rich medium that yields a growth rate approximately twice as high. The average swimming speed of $40\,\mu\mathrm{m\,s^{-1}}$ we obtain is consistent with that reported in a study using similar growth conditions: Turner et al. [12] report $30 \pm 12\,\mu\mathrm{m\,s^{-1}}$ for AW405 cells grown in TB with fluorescently labelled flagella observed in a motility medium similar to the one used by us, except for the presence of 0.18% (hydroxypropyl)methylcellulose in our experiments. The addition of methylcellulose increases the swimming speed by approximately one third [13, 14]. In the absence of methylcellulose, we obtain an average speed matching that reported by Turner et al. [12].

Surprisingly, Molaei et al. [7] find a low swimming speed of $14\,\mu\mathrm{m\,s^{-1}}$ like Berg & Brown [5], despite using a rich growth medium. A potential reason for the discrepancy might lie in the high salt concentration of Molaei et al.'s growth medium (0.5 M, approximating the salinity of the ocean, while we use 0.5% (w/v), corresponding to 86 mM). A severe downregulation of motility for *E. coli* grown in TB with such high NaCl concentrations has been reported [15]. Other differences include composition and pH value of the motility medium used (pH 7.5, whereas our and most other bacterial motility studies use pH 7.0).

We conclude that the *E. coli* swimming speeds we report are consistent with literature values.

References

[1] Butler, J. P. & Reeds, J. A. Stereology of Dihedral Angles I: First Two Moments. *SIAM J. Appl. Math.* **47**, 670–677 (1987).

[2] Locsei, J. T. Persistence of direction increases the drift velocity of run and tumble chemotaxis. *J. Math. Biol.* **55**, 41–60 (2007).

[3] Taktikos, J., Stark, H. & Zaburdaev, V. How the motility pattern of bacteria affects their dispersal and chemotaxis. *PLoS One* **8**, e81936 (2013).

[4] Lovely, P. & Dahlquist, F. Statistical measures of bacterial motility and chemotaxis. *J. Theor. Biol.* **50**, 477–496 (1975).

[5] Berg, H. & Brown, D. Chemotaxis in Escherichia coli analysed by three-dimensional tracking. *Nature* **239**, 500–504 (1972).

[6] Bryant, F. D., Seiber, B. a. & Latimer, P. Absolute optical cross sections of cells and chloroplasts. *Arch. Biochem. Biophys.* **135**, 97–108 (1969).

[7] Molaei, M., Barry, M., Stocker, R. & Sheng, J. Failed Escape: Solid Surfaces Prevent Tumbling of Escherichia coli. *Phys. Rev. Lett.* **113**, 068103 (2014).

[8] Vater, S. M. *et al.* Swimming behavior of Pseudomonas aeruginosa studied by holographic 3D tracking. *PLoS One* **9**, e87765 (2014).

[9] Sheng, J. *et al.* Digital holographic microscopy reveals prey-induced changes in swimming behavior of predatory dinoflagellates. *Proc. Natl. Acad. Sci. U. S. A.* **104**, 17512–17517 (2007).

[10] Molaei, M. & Sheng, J. Imaging bacterial 3D motion using digital in-line holographic microscopy and correlation-based de-noising algorithm. *Opt. Express* **22**, 3232–3238 (2014).

[11] Jaqaman, K. *et al.* Robust single-particle tracking in live-cell time-lapse sequences. *Nat. Methods* **5**, 695–702 (2008).

[12] Turner, L., Ryu, W. & Berg, H. Real-time imaging of fluorescent flagellar filaments. *J. Bacteriol.* **182** (2000).

[13] Berg, H. & Brown, D. Chemotaxis in Escherichia coli analysed by three-dimensional tracking. *Antibiot. Chemother.* **19**, 55–78 (1974).

[14] Darnton, N. C., Turner, L., Rojevsky, S. & Berg, H. C. On torque and tumbling in swimming Escherichia coli. *J. Bacteriol.* **189**, 1756–64 (2007).

[15] Shin, S. & Park, C. Modulation of flagellar expression in Escherichia coli by acetyl phosphate and the osmoregulator OmpR. *J. Bacteriol.* **177**, 4696–4702 (1995).
